# Omega-3 Fatty Acids Weaken Lymphocyte Inflammatory Features and Improve Glycemic Control in Nonobese Diabetic Goto-Kakizaki Rats

**DOI:** 10.3390/nu16234106

**Published:** 2024-11-28

**Authors:** Tiago Bertola Lobato, Elvirah Samantha de Sousa Santos, Patrícia Nancy Iser-Bem, Henrique de Souza Falcão, Gabriela Mandú Gimenes, Janaina Ribeiro Barbosa Pauferro, Glayce Tavares Rodrigues, Ilana Souza Correa, Ana Carolina Gomes Pereira, Maria Elizabeth Pereira Passos, João Carlos de Oliveira Borges, Amara Cassandra dos Anjos Alves, Camila Soares dos Santos, Maria Janaina Leite de Araújo, Vinícius Leonardo Sousa Diniz, Adriana Cristina Levada-Pires, Tânia Cristina Pithon-Curi, Laureane Nunes Masi, Rui Curi, Sandro Massao Hirabara, Renata Gorjão

**Affiliations:** 1 Interdisciplinary Post-Graduate Program in Health Sciences, Cruzeiro do Sul University, São Paulo 01506-000, Brazilruicuri59@gmail.com (R.C.); renata.gorjao@cruzeirodosul.edu.br (R.G.); 2 National Commercial Learning Service (SENAC), São Paulo 01102-000, Brazil; 3 Department of Physiological Sciences, Multicenter Graduate Program in Physiological Sciences, Federal University of Santa Catarina, Florianópolis 88040-900, Brazil; 4 Educantion Center, Butantan Institute, São Paulo 05585-000, Brazil

**Keywords:** docosahexaenoic acid—DHA, eicosapentaenoic acid—EPA, T lymphocytes, regulatory T cells, insulin resistance

## Abstract

**Highlights:**

- Fish oil supplementation reduces insulin resistance in nonobese type 2 diabetes model.
- Fish oil supplementation increased the lymphocyte polarization towards T regulatory profile instead Th1 and Th17 profiles.
- Fish oil immunomodulatory effects indicate a potential effect of omega-3 to reduce inflammatory response in lean type 2 diabetic patients.
- Anti-inflammatory effects of fish oil can contribute for the increased insulin response in nonobese type 2 diabetic individuals.

**Abstract:**

Background/Objectives: Goto-Kakizaki (GK) rats exhibit insulin resistance and type 2 diabetes mellitus (T2DM) without obesity. This study explored the effects of ω-3 fatty acid supplementation on T lymphocyte polarization in Wistar (WT) and GK rats. Methods: They were administered ω-3 fatty acid-rich fish oil (FO) containing eicosapentaenoic (540 mg/g) and docosahexaenoic acids (100 mg/g) by oral gavage at 2 g/kg, thrice a week for 8 weeks. The control groups (WT CT and GK CT) received the same volume of water. The following groups were investigated: GK CT, *n* = 14; GK ω-3, *n* = 15; Wistar CT, *n* = 15; and Wistar ω-3, *n* = 11. Glucose and insulin tolerance tests (GTT and ITT) were performed. Fasting plasma insulinemia and glycemia were measured. After euthanasia, the lymphocytes were extracted from the mesenteric lymph nodes. Results: The results showed that GK rats supplemented with FO had significantly improved glucose tolerance and insulin sensitivity (kITT). It also promoted greater polarization of lymphocytes toward T regulatory (Treg) features and a reduction in Th1 and Th17 profiles. Additionally, the GK ω-3 group exhibited lower cell proliferation, decreased pro-inflammatory cytokines, and increased IL-10 levels compared to the GK control. Conclusions: In conclusion, FO supplementation benefited GK rats by improving glucose intolerance, suppressing insulin resistance, and modulating lymphocytes toward Treg polarization.

## 1. Introduction

Patients with type-2 diabetes mellitus (T2DM) exhibit chronic, low-grade inflammation [1]. This condition is characterized by high plasma levels of inflammatory mediators, such as tumor necrosis factor-α (TNF-α), interleukin (IL)-1, IL-6, IL-8, leptin, monocyte chemotactic protein, resistin, chemokines, C-reactive protein (CRP), and serum amyloid protein as well as low levels of anti-inflammatory cytokines and adipokines such as IL-10 and adiponectin. Inflammation suppresses insulin signaling and causes insulin resistance (IR) leading to metabolic syndrome and T2DM [2,3,4,5,6,7,8,9]. Patients with T2DM exhibit an increase in pro-inflammatory cells such as M1-like macrophages, T helper 1 (Th1), Th17, and CD8+ cells [4,10,11], and a decrease in anti-inflammatory cells, such as M2-like macrophages, Th2 cells, regulatory T cells (Tregs), and IgM-producing B-1 cells [4,12,13,14]. Tregs suppress the Th1-, Th2-, and Th17-associated responses, exacerbating IR. The proportion of Tregs is markedly reduced in newly diagnosed patients with T2DM [15,16]. This inflammatory state also exhibits high levels of pro-inflammatory cytokines and is associated with chronic non-communicable diseases such as T2DM, cardiovascular diseases, and cancer [17,18].

A considerable number of nonobese patients exhibit T2DM, particularly in Japan [19]. The Goto-Kakizaki (GK) rat is a nonobese experimental model of T2DM. GK rats demonstrate fasting hyperglycemia, glucose intolerance, peripheral IR, chronic inflammation, a reduction in the number of pancreatic β cells, a remodeling of the small intestine with localized inflammation, and reduced intestinal transit [20,21,22,23]. These features closely resemble those observed in humans with T2DM, making GK rats an excellent model for studying this disease [24,25,26]. Our group recently reported that lymphocytes from 21-day-old GK rats exhibited diminished Treg lymphocyte markers. At 4 months, GK rats displayed an enhanced Th1 response and reduced GATA-3 expression (crucial for Th2 cell differentiation). These findings highlight an early breakdown of anti-inflammatory mechanisms, indicating pro-inflammatory T lymphocyte features are involved in chronic inflammation reported in GK rats [27].

Long-chain omega-3 (ω-3) fatty acids, such as eicosapentaenoic acid (EPA) and docosahexaenoic acid (DHA), have anti-inflammatory properties [28,29]. The British and American Diabetes Associations recommend EPA and DHA for patients with T2DM, due to the association of these fatty acids with slower T2DM progression. Their ability to modulate the inflammatory response plays a crucial role in preventing and treating IR [30,31,32,33].

EPA and DHA suppress the activation of nuclear factor ƙB (NF-κB), a key signaling molecule in the inflammatory response. This reduction suppresses the production of pro-inflammatory cytokines by human monocytes and is associated with the inhibition of ƙB (IκB) phosphorylation [34,35]. ω-3 fatty acids also elevate the production of inflammation-related resolvins and protectins [36,37]. They decrease lymphocyte proliferation [38] and inhibit the secretion of inflammatory cytokines, such as IL-2, TNF-α, and IFN-γ. In GK rats, they reduce plasma glucose, triglyceride, and cholesterol levels [39,40]. EPA and DHA benefit obese humans and diabetic animals [41,42]. However, it remains unknown if they have the same effects in nonobese T2DM models.

We investigated the effects of FO supplementation on the polarization of T lymphocytes toward an anti-inflammatory phenotype in GK rats. Supplementation with ω-3 fatty acids reduced lymphocyte polarization toward Th1 and Th17 and increased Treg cells indicating anti-inflammatory properties. Thus, it may also be beneficial in nonobese T2DM models.

## 2. Materials and Methods

### 2.1. Animals and Treatment

Adult male WT and GK rats were obtained from Charles River Laboratories International, Inc. (Wilmington, MA, USA). They were housed in the vivarium of the Interdisciplinary Postgraduate Program in Health Sciences at the University of Cruzeiro do Sul, São Paulo, Brazil. They were kept in groups of five rats per cage in a room with a 12 h light/dark cycle, a constant temperature of 22 ± 2 °C, circulating air, and free access to water and food consisting of Nuvilab CR-1 standard rodent diet (Quimtia S/A, Colombo, Paraná, Brazil) throughout the study. Animals at 8 weeks of age were divided into four groups: WT Control (WT CT) (*n* = 15), WT Omega-3 (WT ω-3) (*n* = 11), GK Control (GK CT) (*n* = 14), and GK Omega-3 (GK ω-3) (*n* = 15). The WT ω-3 and GK ω-3 groups received a HiÔmega-3 FO rich in ω-3 fatty acids (Naturalis^®^, Arujá, São Paulo, Brazil), which contained 540 mg of EPA and 100 mg of DHA per g. The dosage of 2 g/kg of body mass, was administered thrice a week over 8 weeks through gavage [43]. The WT CT and GK CT groups received the same volume of water.

The animals were euthanized at 16 weeks with an intraperitoneal injection of ketamine hydrochloride (180 mg/kg of body weight) and xylazine hydrochloride (30 mg/kg of body weight). All procedures in this study adhered to the principles defined by the Cruzeiro do Sul University’s Animal Experimentation Ethics Committee (Protocol Number: 010-2020).

### 2.2. Body Weight, Calculation of the Lee Index, and Caloric Intake

Body mass and food consumption were monitored twice a week throughout the experiment using a Marte AD3300 precision scale (Santa Rita do Sapucaí, Minas Gerais, Brazil). The naso-anal length was measured with a tape measure at euthanasia. The animal body mass index was estimated by calculating the Lee index by applying the formula: ∛ body weight/naso-anal distance [44]. Results < 0.30 were considered normal. Adiposity was determined based on the sum of the retroperitoneal, renal, epididymal, and subcutaneous adipose tissues measured on a Mettler Toledo PB001 precision scale (Barueri, São Paulo, Brazil). Caloric consumption was ascertained by multiplying the food intake results with the calorie count of Nuvilab CR-1.

### 2.3. Glucose Tolerance Test (GTT)

At 15 weeks of age, a GTT was carried out following a 6 h fasting period. The animals were intraperitoneally injected with a 50% glucose solution at 2 g/kg body weight. Blood samples were collected through a cut in the tail tip, and glucose concentration was measured with an Accu-Chek blood glucose monitor (Roche, São Paulo, São Paulo, Brazil) at the regular time points of 0 (pre-injection), 15, 30, and 60 min post-glucose administration. Glucose levels over time were plotted on a graph, and the area under the curve (AUC) for each animal was calculated [23,45].

### 2.4. Insulin Tolerance Test (ITT)

After 2 days of GTTs, an ITT was performed after a 6 h fasting period. Each animal was intraperitoneally injected with insulin (Humulin R; Lilly, Indianapolis, IN, USA) at 0.75 IU/kg of body weight [46]. Blood samples were obtained by cutting the tail tip, and the glucose concentration was determined using an Accu-Chek blood glucose monitor (Roche) at different time intervals: at 0 (pre-injection), 4, 8, 12, 15, and 20 min after insulin administration. The ITT glucose decay constant ratio (kITT) was calculated based on the linear decline in blood glucose levels obtained between 0 and 12 min from the ITT curve [23,45,47].

### 2.5. Determination of Blood Serum Parameters

At the time of euthanasia, the blood of the animals, which had been fasting for 6 h, was collected and the serum was stored at −80 °C. An aliquot of the serum was thawed, and the insulin concentration was assessed using the EZRMI-13K commercial Rat/Mouse Insulin ELISA kit (Merck, Darmstadt, Germany) utilizing the Sandwich ELISA method. The absorbance was read on a Varioeskan LUX Multimode microplate reader (Thermo Fisher Scientific, Waltham, MA, USA), following the manufacturer’s instructions.

The insulin resistance index (HOMA-IR) was calculated by applying the formula: HOMA-IR = (glycemia [mM] × insulin [mU/L])/22.5 [48,49]. In addition, the Quantitative Insulin Sensitivity Check Index (QUICKI) was ascertained utilizing the formula: QUICKI = 1/(log basal glycemia + log basal insulin) [48,50].

For the biochemical analyses of glucose, creatine kinase NAC, lactate dehydrogenase, CRP, total cholesterol, and low-density lipoproteins (LDLs), commercial kits from Kovalent (São Gonçalo, Rio de Janeiro, Brazil) were used. These kits are widely recognized for their accuracy and reliability. The readings were obtained utilizing an automated biochemical analyzer, ensuring standardization and reducing the risk of human error. The K117 Bioclin kit (Belo Horizonte, Minas Gerais, Brazil) was used to measure triglycerides, which were analyzed by employing the enzymatic colorimetric method.

### 2.6. Isolation of Lymphocytes

Lymphocytes were obtained from the mesenteric lymph nodes according to the methodology described previously [27,51,52,53]. The cell population obtained through this procedure consisted of 60% T lymphocytes and 40% B lymphocytes, with less than 1% contamination by macrophages. Cells were counted under a light microscope with a Neubauer chamber. CD3+ lymphocytes were isolated using BD IMagTM anti-CD3 (Reference 554831 and 557812, Becton Dickinson, San Jose, CA, USA) magnetic particles according to the manufacturer’s instructions (Becton Dickinson). These cells were used to analyze gene expression, glucose uptake, and cytokine secretion.

### 2.7. Lymphocytes Culture

Lymphocytes were cultured in RPMI-1640 (Thermo Fisher Scientific, Waltham, MA, USA) medium enriched with 2 mM glutamine, buffered with 24 mM sodium bicarbonate, 20 mM HEPES, 10% fetal bovine serum (FBS), and antibiotics (10,000 U/mL penicillin and 10,000 mg/mL streptomycin). The culture was incubated at 37 °C and under 5% CO_2_ + 95% atmospheric air, per the previous protocol Curi and Peres [54]. The lymphocytes were maintained at 1 × 10^6^ cells per mL for all experiments.

### 2.8. Cell Proliferation Assay

T lymphocyte proliferation was measured using an APC BrdU Flow Cytometry Kit (Catalog number: 8817-6600-42; Thermo Fisher Scientific), as described by Lobato et al. [27]. Briefly, lymphocytes were resuspended in RPMI-1640 medium, stimulated with Concanavalin A (5 μg/mL), and incubated with BrdU (5 μM) for incorporation into the DNA strand for 48 h in culture at 37 °C and an atmosphere of 95% air and 5% CO_2_. The cells were then fixed and permeabilized, followed by treatment with DNase (300 μg/mL) and incubation with the FITC-conjugated anti-BrdU antibody (1:50). After the subsequent washes, the cells were analyzed by flow cytometry on the BD-Accuri, acquiring ten thousand events per sample, and the histograms analyzed using the “BD-C6 Sampler software” (version 1.0.264.21, Becton Dickinson).

### 2.9. RT-PCR

Total RNA was extracted from a population of 1 × 10^7^ total CD3+ T lymphocytes obtained from the mesenteric lymph nodes. The mRNA levels of the genes T-bet, IFN-γ, TNF-α, IL-18, IL-2, GATA-3, IL-4, ROR-γ, TGF-β, IL-17, IL-6, FOXP3, IL-35, IL-10, and Glut-1 were evaluated by RT-PCR (Thermo Fisher Scientific), per the protocol described by Lobato et al. [27].

The following formula was used to calculate the ratio between the transcript and the reference gene (Rplp0): 2^−DDCT^, where DCT is the difference between the CT of the gene of interest and that of the reference, and DDCT is equal to the DCT of the experimental group minus the mean of the DCT of the control group (WT CT) [55,56]. The gene-specific primers were designed by accessing the public Gene Bank database of the National Center for Biotechnology Information (NCBI; http/www.ncbi.nlm.nih.gov/genbank, accessed on 3 February 2022). Their sequences are shown in Table 1.

### 2.10. Lymphocyte Profile Evaluation by Flow Cytometry

Total lymphocytes (1 × 10^6^ cells) were maintained in culture under stimulation with 1 μg/mL phorbol myristate acetate (PMA) and 25 μg/mL ionomycin for 12 h each. Next, the cells were washed with PBS containing 1% BSA and dark-incubated with anti-CD4+ FITC at 1:200 (ab210349; Abcam, Cambridge, UK) for 30 min. They were then fixed with 4.2% formaldehyde for 30 min and dark-incubated with BD GolgiStop™ (554724; Becton Dickinson) for 30 min. After washing, the following antibodies were added: anti-TNF-α (Th1, ab6671; Abcam), anti-IL-4 (Th2, ab9811; Abcam), anti-ROR-γ-PE (Th17, 12-6988-82; Thermo Fisher Scientific), and anti-FOXP3-PerCP-Cyanine5.5 (Treg, 45-5773-82; Thermo Fisher Scientific). They were dark-incubated for 30 min. For Th1 and Th2 analyses, we treated cells with anti-IgG Alexa 647 (ab150159; Abcam). For Th17 and Treg analyses, we proceeded to washing as described before. Isotype controls were used for each fluorochrome. After washing, 20,000 positive CD4+ events were acquired using flow cytometry and analyzed utilizing the BD Accuri software (version 1.0.264.21, Becton Dickinson).

### 2.11. Expression of CD28 on Lymphocyte Membrane

The expression of CD28 on the lymphocyte surface was assessed by flow cytometry. The cell suspension containing 1 × 10^6^ lymphocytes was washed with PBS containing 1% BSA and incubated with anti-CD28-PE antibody (ab176502; Abcam) at a dilution of 1:20. After incubation, the cells were rewashed with PBS containing 1% BSA and examined by flow cytometry, where 10,000 events were acquired in the histograms and analyzed with the BD C6 Sampler™ software (Becton Dickinson). Isotype controls were used for each fluorochrome.

### 2.12. Glucose Uptake

CD3+ T lymphocytes (1 × 10^6^ cells/mL) were initially incubated with 1 μg/mL PMA and 25 μg/mL ionomycin in RPMI 1.640 medium supplemented with 10% FBS. Glucose uptake was ascertained using a glucose uptake cell-based assay kit (Catalog number 600470; Cayman, Ann Arbor, MI, USA), according to the manufacturer’s instructions. The lymphocytes were incubated with 20 μM 2-NBDG (fluorescence-labeled deoxyglucose analog) at 37 °C for 60 min. Subsequently, the cells were analyzed utilizing the BD Accuri flow cytometer (Becton Dickinson) and an FL1 filter. Unlabeled controls were generated by incubating the cells, as described earlier, but in the absence of 2-NBDG. A total of 10,000 events were recorded, and the mean fluorescence for 2-NBDG (in FL-1) was determined using side-scatter (SSC) and forward-scatter (FSC) fluorescence and analyzed by employing the BD Accuri software (Becton Dickinson).

### 2.13. Measurement of Cytokine Concentrations in the Lymphocyte Culture Supernatant

After 12 h of T lymphocyte culture (1 × 10^6^ cells/mL) under stimulation with 25 μg/mL PMA and 1 μg/mL ionomicin, the levels of IL-10, IFN-γ, TNF-α, IL-17A, and IL-17F cytokines in the culture supernatant were ascertained using the LEGENDplex™ Rat Th Cytokine Panel kit (Catalog number 741220; BioLegend, San Diego, CA, USA), following the manufacturer’s instructions. Initially, the samples were diluted 1:4 in the assay buffer. The entire assay was carried out in a V-bottom plate, to which 12.5 µL of assay buffer was added. Then, 12.5 µL of the samples was added to the respective wells, followed by 12.5 µL of the premixed beads. The mixture was then dark-incubated for 2 h at room temperature. The beads were washed to remove unbound antibodies, resuspended in a wash buffer, and analyzed using BD C6 Sampler™ (BD Biosciences). The concentrations were determined using LEGENDplex™ Data Analysis Software (version B391015, BioLegend).

### 2.14. Statistical Analysis

The normality of the data distribution was assessed utilizing the Shapiro-Wilk normality test. Intergroup comparisons were performed using a repeated-measures analysis of variance (Two-Way ANOVA). In situations where significant differences were observed, post-hoc analyses employed Sidak’s post-test adjustment. The level of statistical significance adopted was *p* ≤ 0.05. All analyses were performed using the GraphPad Prism software version 8.0 (GraphPad, San Diego, CA, USA). The results are expressed as the means ± standard errors.

## 3. Results

### 3.1. Body Weight, Lee Index, Caloric Intake, GTT, and ITT

**Figure 1:**
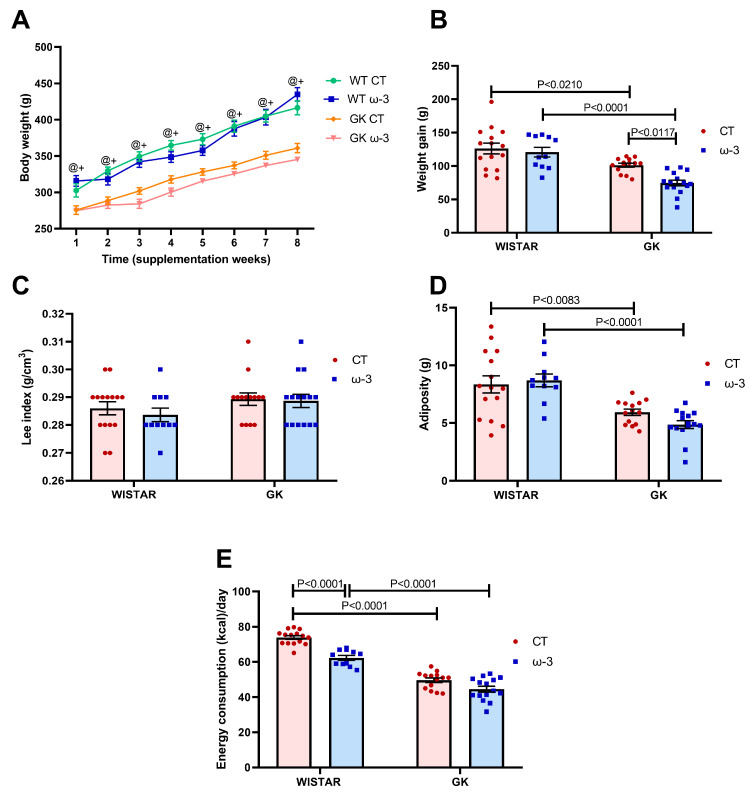
Evaluation of body weight during the 8 weeks of the experiment (**A**), body weight gain (final–initial) (**B**), Lee Index (**C**), adiposity (**D**), and energy consumption (Kcal/day) (**E**). WT CT, (*n* = 15); WT ω-3, (*n* = 11); GK CT, (*n* = 14); and GK ω-3 (*n* = 15). ^+^ *p* < 0.05 GK CT vs. WT CT; ^@^ *p* < 0.05 GK ω-3 vs. WT ω-3. The results are expressed as the means ± standard errors of the means (SEM). WT, Wistar; CT, control; GK, Goto-Kakizaki.

During the 8 weeks of experimentation, the body weight of the GK CT and GK ω-3 animals was lower compared to the WT groups (Figure 1A,B). The total weight gain in the GK group rats was lower than that in WT rats. FO supplementation lowered weight gain in the GK ω-3 group compared to the GK CT group (Figure 1B). There were no variations in the Lee index (Figure 1C). Regarding adiposity (Figure 1D), GK animals demonstrated lower values than the WT group rats. Concerning daily caloric intake, GK CT and GK ω-3 animals consumed fewer calories than the WT group. The WT ω-3 group had a lower daily caloric intake than its control group (Figure 1E).

**Figure 2:**
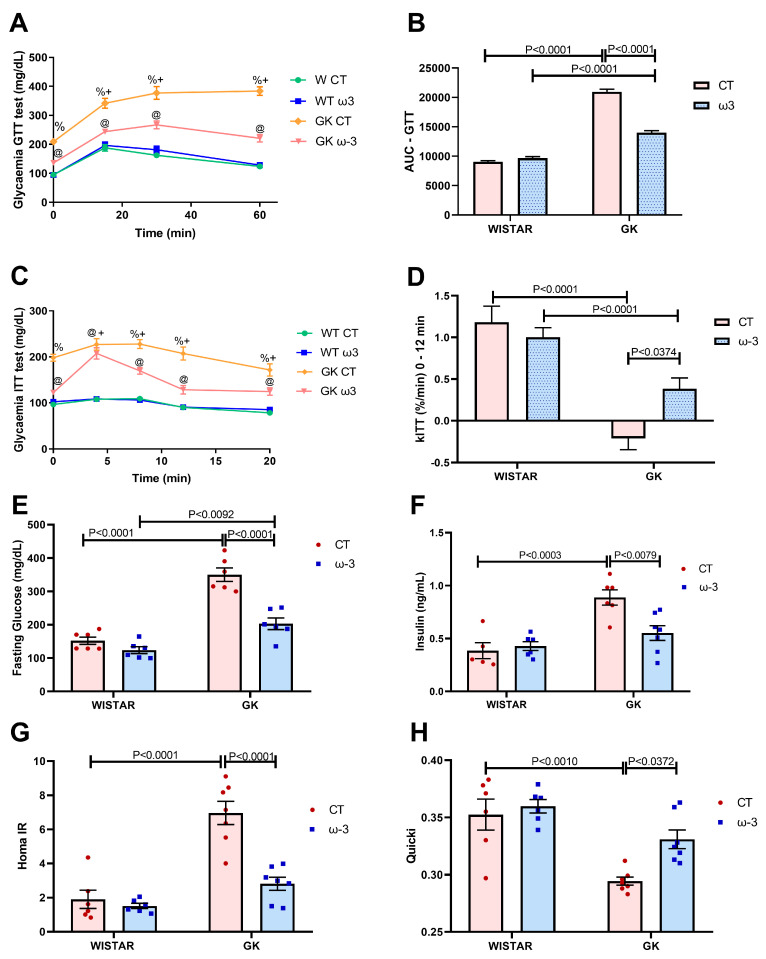
Glucose tolerance test (GTT) (**A**), the area under the GTT curve (**B**), insulin tolerance test (ITT) (**C**), glucose decay constant for ITT (kITT) (**D**), fasting blood glucose (**E**), insulin (**F**), Homa-IR Index (**G**), and QUICKI Index (**H**). For tests A, B, C, and D, we used WT CT, (*n* = 15); WT ω-3, (*n* = 11); GK CT (*n* = 14); and GK ω-3 (*n* = 15). For test E, we used Wistar CT, (*n* = 6); Wistar ω-3, (*n* = 6); GK CT, (*n* = 6); and GK ω-3 (*n* = 6). For tests F, G, and H, we used Wistar CT, (*n* = 6); Wistar ω-3, (*n* = 6); GK CT, (*n* = 7); and GK ω-3 (*n* = 7). ^+^ *p* < 0.05 GK CT vs. Wistar CT, ^@^ *p* < 0.05 GK ω-3 vs. Wistar ω-3, *^%^* GK ω-3 vs. GK CT. The results are expressed as the means ± standard errors of the means (SEM). AUC, Area under curve. WT, Wistar; CT, control; GK, Goto-Kakizaki; HOMA, homeostatic model assessment; kITT, insulin tolerance test rate constant.

Concerning the GTT AUC (Figure 2A,B), the GK CT group exhibited higher values than the WT CT and GK ω-3 groups, whereas GK ω-3 rats had greater values than the WT ω-3 group. In the ITT (Figure 2C,D), WT rats revealed an enhanced glycemic reduction compared to the GK rats as observed in the kITT independent of FO supplementation. GK ω-3 animals had elevated kITT compared to the GK CT group. When analyzing fasting glucose levels, GK CT had markedly higher values than WT CT and GK ω-3 rats (Figure 2E). The fasting plasma insulin, HOMA-IR (an indicator of IR) and QUICKI Index (an indicator of insulin sensitivity) values were enhanced in GK CT compared to the WT CT rats. FO reduced these values in GK rats (Figure 2F–H).

**Figure 3:**
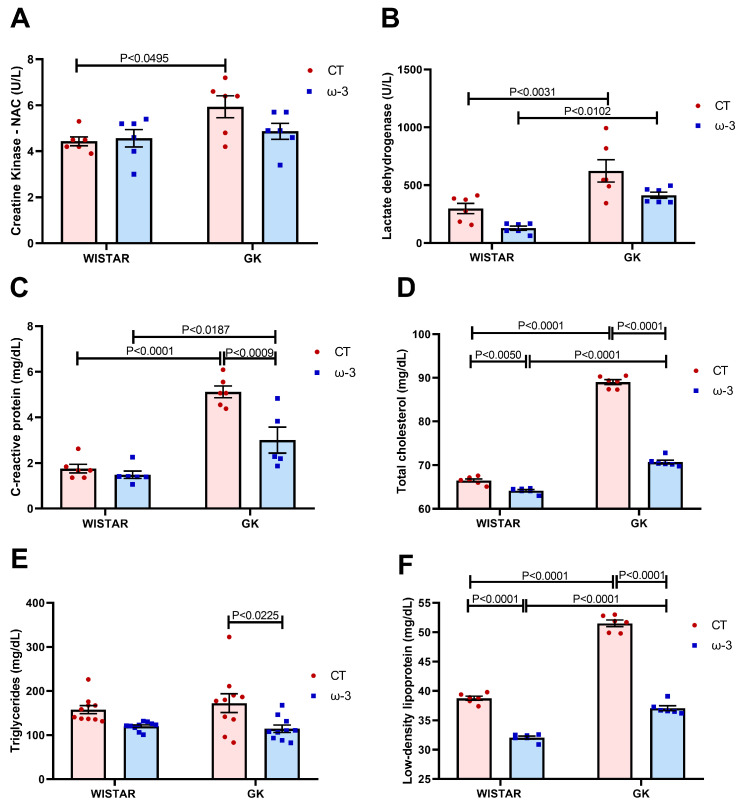
Evaluation of creatinine kinase (**A**), lactate dehydrogenase (**B**), C-reactive protein (**C**), total cholesterol (**D**), triglycerides (**E**), and low-density lipoproteins (**F**). For tests A, B, C, D, and F, we used Wistar CT, (*n* = 6); Wistar ω-3, (*n* = 6); GK CT, (*n* = 6); and GK ω-3, (*n* = 6). For test E, we used Wistar CT, (*n* = 10); Wistar ω-3, (*n* = 10); GK CT (*n* = 10); and GK ω-3 (*n* = 10). The results are expressed as the means ± standard errors of the means (SEM). CT, control; GK, Goto-Kakizaki; NAC, N-Acetylcysteine.

The activities of serum creatine kinase were higher in the GK CT than in the WT CT group animals (Figure 3A). Lactate dehydrogenase serum activity was enhanced in the GK group compared to the WT group (Figure 3B). CRP levels were elevated in the GK group compared to the WT group, and ω-3 fatty acid supplementation reduced them in GK rats (Figure 3C).

Total cholesterol and LDL were higher in the GK compared to the WT groups (Figure 3D,F). However, supplementation with ω-3 suppressed these values for both GK and WT groups compared to the control ones. Triglycerides declined in the GK ω-3 group when compared to the GK CT group (Figure 3E).

### 3.2. Lymphocyte Inflammatory Features

**Figure 4:**
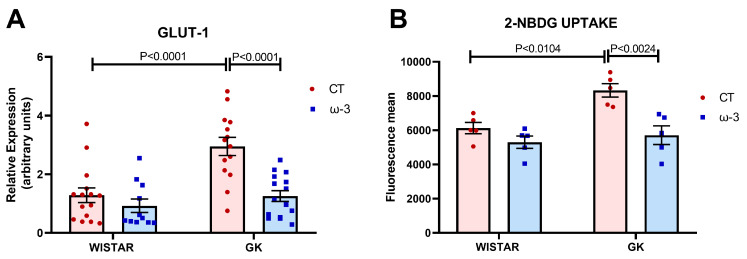
Glucose Transporter 1 (GLUT-1) mRNA expression (**A**) and 2-NBDG uptake under PMA and ionomycin stimulation (**B**). GLUT1 expression was evaluated by real-time PCR. 2^−DDCT^ was used to calculate the relationship between the transcripts and the reference gene (*RPLP0*). Statistical analysis of the mean fluorescence intensity related to 2-NBDG incorporation in lymphocytes is shown in B. For test A, we used Wistar CT (*n* = 15); Wistar ω-3, (*n* = 11); GK CT, (*n* = 14), and GK ω-3, (*n* = 15). For test B, we used Wistar CT (*n* = 5), Wistar ω-3, (*n* = 5) GK CT, (*n* = 5), and GK ω-3, (*n* = 5). The results are expressed as the means ± standard errors of the means (SEM). PMA, 12 h with phorbol myristate acetate; CT, control; GK, Goto-Kakizaki; 2-NBDG; 2-(N-(7-Nitrobenz-2-oxa-1,3-diazol-4-yl)Amino)-2-Deoxyglucose.

The T lymphocytes of the control GK animals showed an increased expression of *GLUT-1* compared to the WT CT and ω-3-supplemented GK groups (Figure 4A). The GK control T lymphocytes exhibited an increased 2-NBDG fluorescence mean compared to the WT CT and GK ω-3 groups (Figure 4B). These data indicate that glucose consumption by lymphocytes was augmented in GK rats but was abolished by ω-3 fatty acid supplementation. The representative histograms of the 2-NBDG uptake analysis are presented in S3.

**Figure 5:**
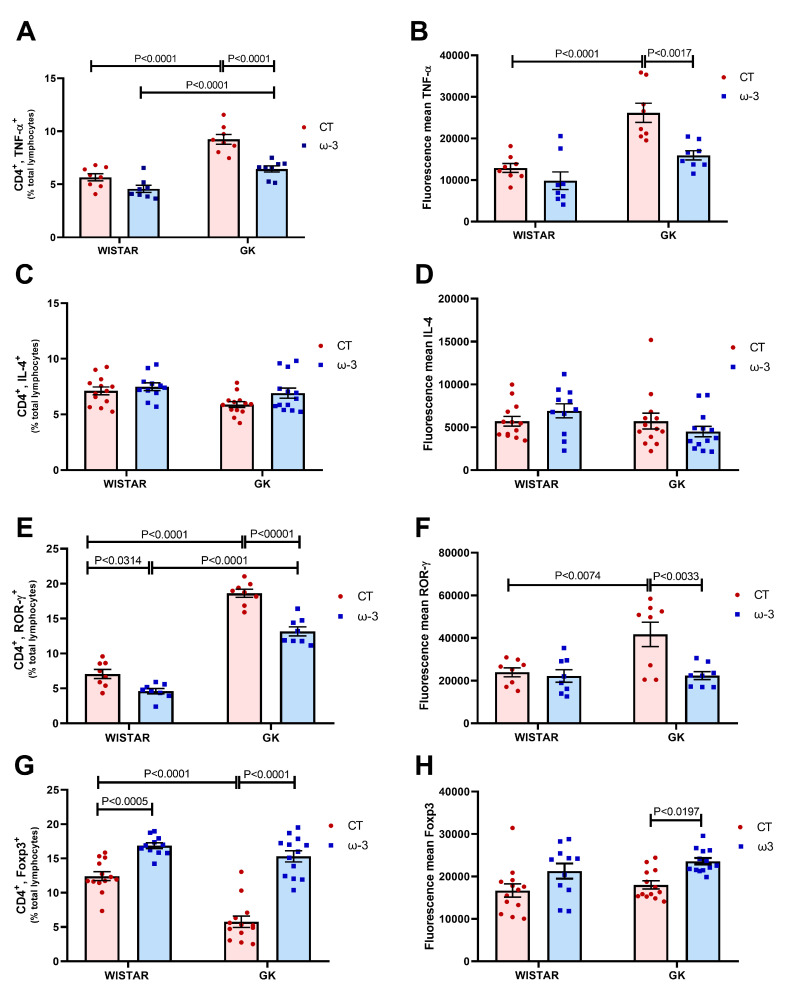
The cells were stimulated with PMA and ionomycin for 12 h to evaluate the T lymphocyte phenotype using flow cytometry. (**A**) Th1 percentage (CD4+TNF-α+ cells); (**B**) TNF-α fluorescence mean; (**C**) Th2 percentage (CD4+IL-4+); (**D**) IL-4 fluorescence mean; Th17 percentage (CD4+ROR-γ+ cells) (**E**); (**F**) ROR-γ fluorescence mean; (**G**) Treg percentage (CD4+FOXP-3+ cells); (**H**) FOXP-3 Fluorescence mean. For tests A, B, E, and F, we used Wistar CT, (*n* = 8); Wistar ω-3, (*n* = 8); GK CT, (*n* = 8); and GK ω-3, (*n* = 8). For tests C, D, G, and H, we used Wistar CT, (*n* = 13); Wistar ω-3, (*n* = 11); GK CT, (*n* = 13); and GK ω-3, (*n* = 13). The results are expressed as the means ± standard errors of the means (SEM). PMA, 12 h with phorbol myristate acetate; CT, control; GK, Goto-Kakizaki; FOXP3, Forkhead box P3; ROR-γ, RAR-related orphan receptor-γ; TNF-α, tumor necrosis factor-α; CD, Cluster of Differentiation.

The flow cytometry evaluation of the T lymphocyte profile upon PMA and ionomycin stimulation revealed the following:

- **Th1 lymphocytes:** The percentage of CD4+TNF-α+ cells in the GK CT group was higher compared to the WT CT and GK ω-3 groups. However, the GK ω-3 group exhibited a lower proportion of Th1 cells than the WT ω-3 group (Figure 5A). Additionally, the mean fluorescence intensity vital for the intracellular detection of TNF-α was higher in the GK CT group compared to the WT CT and GK ω-3 groups (Figure 5B).
- **Th2 lymphocytes:** There were no remarkable variations in the percentage of CD4+ IL-4+ cells or the mean fluorescence intensity related to IL-4 (Figure 5C,D).
- **Th17 lymphocytes:** The proportion of CD4+ ROR-γ+ cells was enhanced in the GK CT group compared to the WT CT and GK ω-3 groups. However, the WT ω-3 group had a lower percentage compared to the WT CT, while the GK ω-3 group had a higher percentage compared to the WT ω-3 group (Figure 5E). The mean fluorescence intensity for ROR-γ was also elevated in the GK CT group compared to the WT CT and GK ω-3 groups (Figure 5F).
- **Treg lymphocytes:** A lower percentage of CD4+ FOXP-3+ cells was observed in the GK CT group compared to the WT CT and GK ω-3 groups (Figure 5G). However, their proportion was enhanced in the WT ω-3 group compared to the WT CT group. In terms of mean fluorescence intensity for FOXP-3, the GK ω-3 group demonstrated a higher value compared to the GK CT group (Figure 5H).

A figure showing the gating strategy of the flow cytometry analysis is represented in Supplementary Materials (S1). The representative dot-plots and histograms are presented in S2.

**Figure 6:**
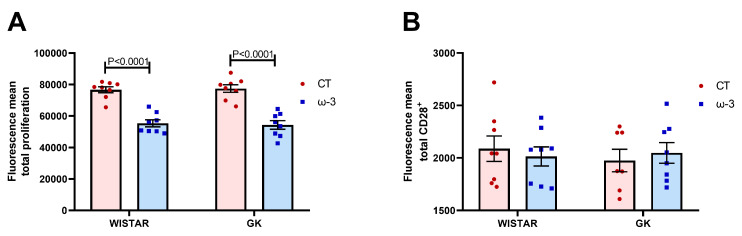
Proliferative capacity of the lymphocytes stimulated with concanavalin A (**A**). CD28+ expression (**B**). For the analysis of cell proliferation, T lymphocytes were stimulated with 5 μg/mL concanavalin A and incubated with 5 μM BrdU for 48 h. Statistical analysis of the mean fluorescence intensity is shown in B. Wistar CT, (*n* = 8); Wistar ω-3, (*n* = 8); GK CT (*n* = 8); and GK ω-3, (*n* = 8). The results are expressed as the means ± standard errors of the means (SEM). CT, control; GK, Goto-Kakizaki; CD, Cluster of Differentiation.

FO supplementation inhibited lymphocyte proliferative capacity in both WT and GK rats (Figure 6A).

CD28 expression did not differ between the groups (Figure 6B). The representative histograms of these analyses are presented in S3.

**Figure 7:**
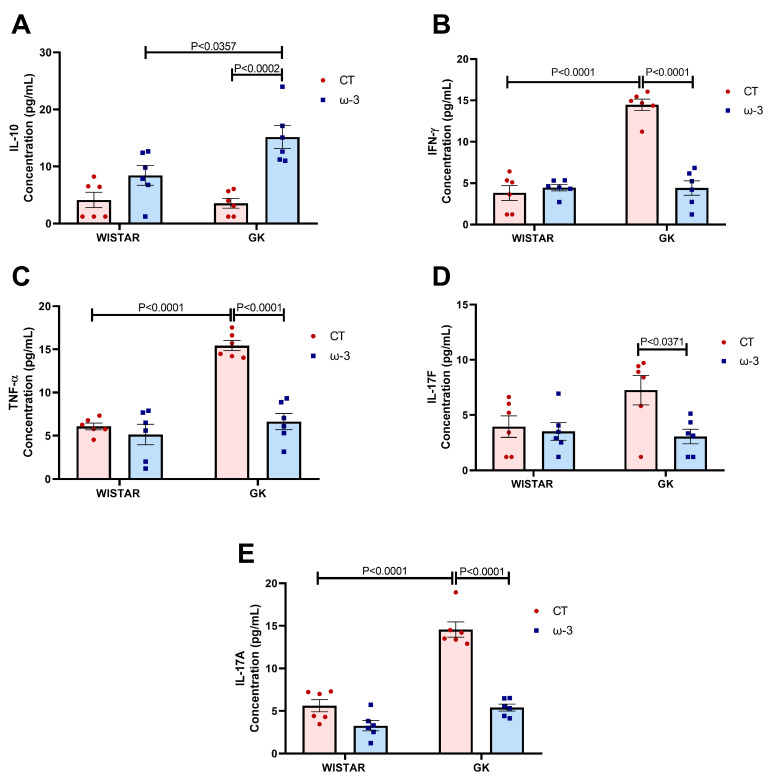
Cytokine concentrations in the supernatant of the T lymphocyte cultures in the presence of PMA and ionomycin. (**A**) IL-10, (**B**) IFN-γ, (**C**) TNF-α, (**D**) IL-17F, and (**E**) IL-17A concentrations. Wistar CT, (*n* = 6); Wistar ω-3, (*n* = 6); GK CT, (*n* = 6); and GK ω-3, (*n* = 6). The results are expressed as the means ± standard errors of the means (SEM). CT, control; GK, Goto-Kakizaki; PMA, 12 h with phorbol myristate acetate; IL, interleukin; TNF-α, tumor necrosis factor-α; IFN-γ, interferon-γ.

The secretion of IFN-γ, TNF-α, and IL-17A by lymphocytes was higher in GK CT compared to WT CT rats. IFN-γ, TNF-α, IL-17F, and IL-17A declined in the GK ω-3 group compared to GK CT group (Figure 7B–E). IL-10 was elevated in the GK ω-3 group compared to the GK CT and WT ω-3 groups (Figure 7A).

**Figure 8:**
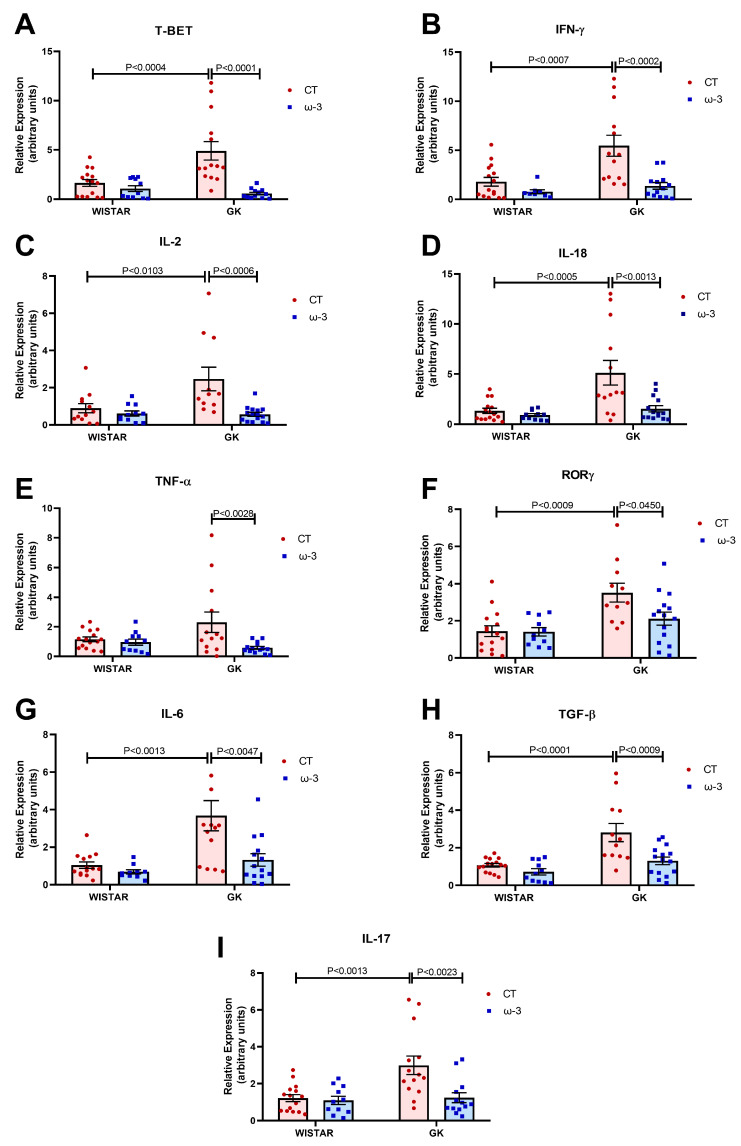
Evaluation of the expression of genes associated with the Th-1 and Th-17 lymphocyte profile in isolated CD3+ lymphocytes. (**A**) T-bet, (**B**) IFN-γ, (**C**) IL-2, (**D**) IL-18, (**E**) TNF-α, (**F**) ROR-γ, (**G**) IL-6, (**H**) TGF-β, and (**I**) IL-17. Gene expression was analyzed by using real-time PCR and the 2^−ΔΔCT^ method. *RPLP0* was used as a constitutive internal control to normalize the data. For test A and I, we used Wistar CT, (*n* = 15); Wistar ω-3, (*n* = 11); GK CT, (*n* = 14); and GK ω-3, *(n* = 13). For tests C and E, we used Wistar CT, (*n* = 15); Wistar ω-3, (*n* = 11); and GK CT, (*n* = 13). For test D, we used Wistar CT, (*n* = 15); Wistar ω-3, (*n* = 9); GK CT, (*n* = 13); and GK ω-3, (*n* = 13). For test E, we used Wistar CT, (*n* = 12); Wistar ω-3, (*n* = 11); GK CT, (*n* = 11); and GK ω-3, (*n* = 15). For tests F and H, we used Wistar CT, (*n* = 15); Wistar ω-3, (*n* = 11); GK CT, (*n* = 12); and GK ω-3, (*n* = 15). For test G, we used Wistar CT, (*n* = 14); Wistar ω-3, (*n* = 10); GK CT, (*n* = 14); and GK ω-3, (*n* = 14). The results are expressed as the means ± standard errors of the means (SEM). CT, control; GK, Goto-Kakizaki; IL, interleukin; IFN-γ, interferon-γ; T-bet, T-box transcription factor; ROR-γ, RAR-related orphan receptor-γ; TGF-β, Transforming growth factor β.

Concerning the expression of genes encoding proteins associated with the inflammatory profile of Th1 cells, we observed that the GK control group exhibited an increased expression of the transcription factor T-bet (Figure 8A), as well as IFN-γ (Figure 8B), IL-18 (Figure 8D), and IL-2 (Figure 8C) genes compared to the WT control group. Upon ω-3 supplementation, the expression of these genes was reduced in the GK group compared to the control. The expression of the TNF-α gene (Figure 8E) was reduced in the GK ω-3 group compared to the GK control group. When assessing the gene expression of the Th17 (inflammatory) profile, the GK control group demonstrated an enhanced expression of the transcription factor ROR-γ (Figure 8F), as well as IL-6 (Figure 8G), TGF-β (Figure 8H), and IL-17 (Figure 8I), when compared to the WT control group. FO supplementation suppressed the expression of these genes in the GK group compared to GK CT.

**Figure 9:**
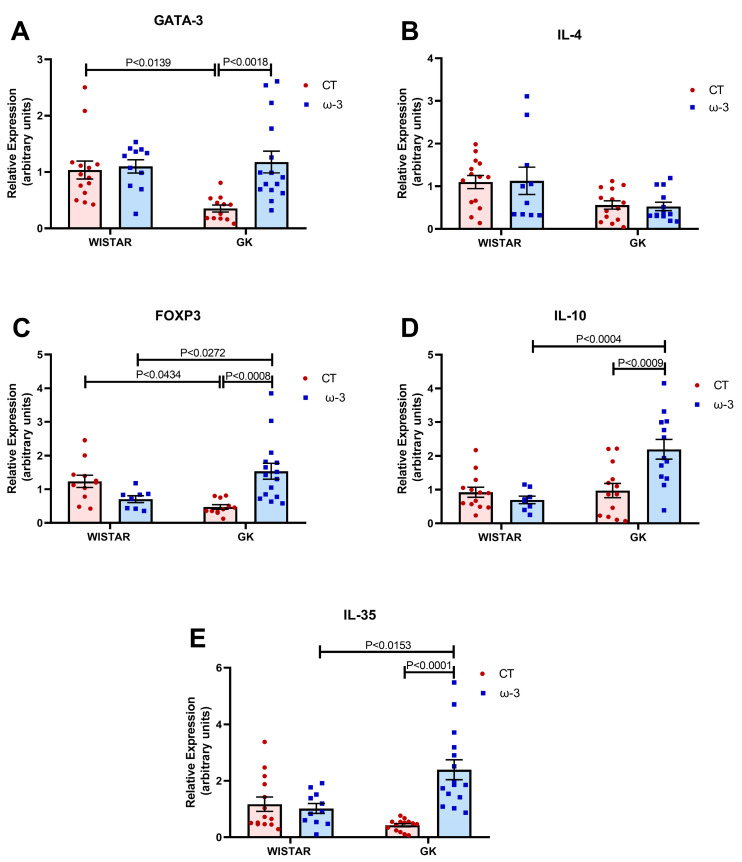
Evaluation of the expression of genes associated with the Th-2 and T regulatory lymphocyte profile in isolated CD3+ lymphocytes. (**A**) GATA-3 and (**B**) IL-4, (**C**) FOXP-3, (**D**) IL-10, and (**E**) IL-35. Gene expression was analyzed using real-time PCR and the 2^−ΔΔCT^ method. RPLP0 was used as a constitutive internal control to normalize the data. For test A, we used Wistar CT, (*n* = 14); Wistar ω-3, (*n* = 11); GK CT, (*n* = 12); and GK ω-3, (*n* = 15). For test B, we used Wistar CT, (*n* = 14); Wistar ω-3, (*n* = 10); GK CT, (*n* = 14); and GK ω-3, (*n* = 13). For test C, we used Wistar CT, (*n* = 11); Wistar ω-3, (*n* = 8); GK CT, (*n* = 13); and GK ω-3, (*n* = 15). For test D, we used Wistar CT, (*n* = 13); Wistar ω-3, (*n* = 8); GK CT, (*n* = 13); and GK ω-3, (*n* = 13). For test E, we used Wistar CT, (*n* = 14); Wistar ω-3, (*n* = 11); GK CT, (*n* = 14); and GK ω-3, (*n* = 15). The results are expressed as the means ± standard errors of the means (SEM). CT, control; GK, Goto-Kakizaki; GATA, GATA binding protein 3; FOXP3, Forkhead box P3; IL, interleukin.

The expression of genes related to the Th2 lymphocyte profile was also assessed. An elevation in the expression of the transcription factor GATA-3 was identified in the GK ω-3 group compared to the GK control (Figure 9A). No significant difference was observed for IL-4, a cytokine released by these cells, between the groups (Figure 9B). Regarding genes associated with the Treg profile, FOXP3 expression (Figure 9C) was lower in the GK control group compared to the WT control animals. FO supplementation enhanced its expression in the GK group, with levels higher than those reported in the WT ω-3 group. Although IL-10 (Figure 9D) and IL-35 (Figure 9E) expression in the GK control group did not vary from that in the WT control group, FO supplementation markedly elevated the expression of these cytokines in the GK group compared to the GK control.

## 4. Discussion

FO rich in ω-3 fatty acids promotes immunomodulation in a nonobese T2DM model. Supplementing GK rats with ω-3 improved the diabetogenic (fasting glucose, insulin, HOMA index, and QUICKI test) and plasma (CRP, total cholesterol, triglycerides, and LDL) factors. Additionally, it positively impacted lymphocyte profiles by shifting the immune response from pro-inflammatory to regulatory characteristics. Specifically, there was an enhancement in CD4+ Treg lymphocytes and a reduction in Th1 and Th17 profiles. This shift in lymphocyte populations was associated with the suppressed expression of inflammatory cytokines and an elevated regulatory immune response. Our findings are consistent with those of Radosinska et al. [40], who observed similar benefits in GK rats complemented with 40 mg/100g/day of ω-3 fatty acids for 8 weeks, and Andersen et al. [57], who reported reduced fasting glycemia and improved insulin sensitivity in diabetic WT rats given 500 mg/kg b.w./day of ω-3 fatty acids for 8 weeks.

The body weight of GK rats supplemented with ω-3 fatty acids was lowered despite no changes in daily caloric intake, Lee’s index, or adiposity, indicating that the influence of ω-3 may occur independently of caloric intake. The unaltered caloric intake suggests that other mechanisms, possibly specific metabolic processes or adipogenesis modulation, may be at play, highlighting the complexity of ω-3 interactions with lipid metabolism. ω-3 regulates lipid metabolism by promoting fatty acid oxidation, suppressing lipogenesis, and activating genes associated with mitochondrial and peroxisomal fatty acid oxidation in the liver and muscles, favoring lipid profile and adipocyte metabolism [58].

We measured serum creatine kinase (CK) and lactate dehydrogenase (LDH) activity, alongside other biochemical markers such as cholesterol and fractions, to assess animal tissue damage and metabolic status. Evaluating CK and LDH in diabetes is crucial, as chronic hyperglycemia can cause tissue damage, increasing the activities of CK and LDH in the blood. Monitoring these markers in diabetics thus aids in assessing potential tissue damage linked to impaired glycemic control or complications [59]. ω-3 fatty acid supplementation did not change these markers. Additionally, the ω-3 fatty acid supplementation could improve plasma lipid features such as total cholesterol and its fractions (LDL, HDL) and triglycerides, as reported by some researchers in obese diabetic conditions. These changes are significant in diabetes, given the elevated heart disease risk associated with cholesterol imbalances [60]. Monitoring the mentioned lipids thus helps assess ω-3′s effectiveness on cardiovascular health.

Examining the effect of ω-3 fatty acids on CRP in individuals with diabetes is highly relevant, as CRP is an inflammation marker associated with elevated cardiovascular risk, particularly in diabetic patients. ω-3 fatty acids have anti-inflammatory properties that may help reduce CRP levels, aiding in decreasing chronic inflammation commonly found in diabetics [61]. This effect may also be associated with changes in lymphocyte profile.

The impact of T2DM on lymphocytes, which polarize toward a pro-inflammatory profile, is well documented [27,62,63,64]. A recent study presented an overall picture of GK rats outside the pancreatic islets [65], demonstrating that circulating CD4+ cells did not differ significantly when compared to the control WT rats. However, GK rats showed a higher proportion of circulating CD8+ cells when compared to the WT control ones. Proportionally, in this study, a marked positive correlation of CD4+/CD8 cells was found but no differences were observed in the Th1/Th2 and Th17/Treg correlations. However, a previous study from our group reported that in lymphocytes isolated from GK rats, lymph nodes exhibited lower T-regulatory lymphocyte markers at 21 days but higher Th1 markers and reduced GATA-3 expression (crucial for Th2 cell differentiation) at 120 days. These findings highlight an early disruption of anti-inflammatory mechanisms in GK rats, indicating a pro-inflammatory profile in lymphocytes that might exacerbate chronic inflammation in T2DM [27]. In the present study, the lymphocytes of GK rats demonstrated a characteristic inflammatory profile, evidenced by a greater polarization of Th1 and Th17 cells, as well as an enhancement in gene expression and secretion of pro-inflammatory cytokines associated with these profiles. FO supplementation reversed this profile, indicating a remarkable anti-inflammatory effect. The underlying mechanism for the reversal of Th1 polarization may be through the modulation of T-bet. It amplifies the IFN-γ response by inducing Th1 cells to release IFN-γ. These findings align with the existing literature highlighting the role of T-bet in regulating inflammatory cytokines such as IFN-γ, TNF-α, IL-18, and IL-2 and its reduction in inflammation [66,67,68,69].

The impact of FO supplementation on Th17 polarization may involve RORγ. Upon activation by TGF-β, it plays a crucial role in the differentiation of lymphocytes to the Th17 profile, promoting the release of pro-inflammatory cytokines such as IL-17 and IL-6 [70]. The observed concomitant reduction in the expression of these cytokine-encoding genes, both at the genetic level and in the supernatant dosages of the IL-17A/F lymphocytes, indicates a potential attenuation of the Th17 inflammatory response by ω-3.

Omega-3 fatty acids, particularly through their bioactive derivatives known as resolvins, markedly impact Th17 lymphocytes, which are a subset of T cells involved in immune response and inflammation [71]. Resolvins interact with cellular signaling pathways that regulate the immune response. They can interfere with signaling pathways such as those involving NF-κB and STAT3, which are crucial for the activation and functioning of Th17 lymphocytes [71].

The balance established between Th17 and Treg subpopulations, which represent two distinct CD4+ T cell phenotypes with completely different functions, is crucial for preventing immune imbalance, autoimmune responses, and pathogenesis of the metabolic syndrome [16]. In fact, supplementation with ω-3 fatty acids affects Treg cells, which are characterized by the expression of CD4+ and CD25+ on the membrane surface and the expression of the transcription factor FOXP3, which plays a critical role in regulating the immune response. These observations suggest that ω-3 fatty acids may promote a more regulatory and suppressive immune response by positively modulating the Tregs [72].

The Th cell profile results suggest that ω-3 fatty acid supplementation reversed the pro-inflammatory CD4+ T lymphocyte profile in GK rats. This effect was further enhanced by an increase in Tregs and a reduction in both Th17 (ROR-γ percentage and mean fluorescence) and Th1 profiles (IFN-γ expression and TNF-α fluorescence). However, ω-3 fatty acids supplementation did not affect the Th1 profile to the extent it did with the Th17 and Treg profiles. This observation indicates that ω-3 fatty acids shifted the lymphocyte polarization from an inflammatory to a regulatory profile. The modulation of Tregs by ω-3 fatty acids is notable, highlighting its impact on the regulatory lymphocyte pathway. This finding supports the hypothesis that this pathway is underutilized and compromised in lean GK rats. Flow cytometry data for Th1, Th2, Th17, and Treg cell profiles were confirmed by gene expression analyses.

Jolly et al. [73] observed that ω-3 fatty acids suppressed Th1 responses by modulating gene expression to lower the production of pro-inflammatory cytokines, interfering with Toll-like receptor signaling, producing anti-inflammatory lipid mediators, and reducing NF-κB activity in mice. These combined impacts inhibited Th1 responses and promoted a more regulatory immune profile. However, the effects of ω-3 fatty acids on lymphocytes in T2DM without obesity may involve additional mechanisms.

This improvement in immune regulation may be linked to the impacts of ω-3 on PPAR (Peroxisome proliferator-activated receptors) transcription factors, particularly PPARα in the liver and PPARγ in the adipose tissue and inflammatory cells. These receptors play a pivotal role in enhancing insulin sensitivity and reducing inflammation [74]. Moreover, ω-3 supplementation boosts PPAR expression across various tissues, which enhances insulin sensitivity and glucose uptake but reduces triglycerides through higher lipoprotein lipase activity and lowers apolipoprotein C3 expression [75].

Metabolites derived from ω-3 fatty acids not only inhibit the differentiation of CD4+ cells into Th1 and Th17 but also suppress the secretion of various cytokines from T cells such as IFN-γ, IL-17, and IL-2 in humans. Predominantly, the ω-3 fatty acid class, with EPA being the most potent, reduces IL-2 production, an impact linked to its inhibition of lymphocyte proliferation and the expression of the IL-2 receptor, resulting in a decrease in virgin T cells and an increase in memory T cells [76,77,78]. In our findings, a decline in proliferation was observed in the ω-3 fatty acid groups.

When T cells recognize antigens via the T cell receptor and receive a second signal via CD28, they increase glycolysis, GLUT1 expression, and glucose uptake to meet their energy and biosynthetic needs, thereby promoting their proliferation, survivability, and capacity for producing effector cytokines such as IFN-γ, IL-17, IL-21, and IL-9 [79]. In addition, the autocrine secretion of IL-2 elevates the expression of GLUT1 and increases the activity of the PI3K pathway [80,81]. Our study found a decrease in GLUT-1 expression and glucose uptake by lymphocytes in ω-3 fatty acid-supplemented groups. However, this did not alter the CD28 cell-surface molecule, which was accompanied by a decline in the expression of IL-2.

Our findings underline the significant role of ω-3 fatty acids in modulating immune responses by shifting lymphocyte profiles from pro-inflammatory to regulatory states. The observed decrease in *GLUT-1* and *IL-2* expression as well as glucose uptake, suggests that ω-3 fatty acid supplementation can influence metabolic pathways essential for T cell activation and function. Despite no changes in the CD28 cell-surface molecule, the overall impact on cytokine profiles and glucose metabolism reinforces the potential of ω-3 fatty acids as a therapeutic strategy to mitigate inflammation in T2DM. Future studies may explore the precise mechanisms through which ω-3 fatty acids exert these effects, providing deeper insights into their role in immune modulation and metabolic regulation in diabetic conditions.

Although this study presents crucial findings on the impacts of ω-3 fatty acids in modulating lymphocyte profiles in a nonobese T2DM model, some limitations must be recognized. The results for the gene expression analysis, glucose consumption, and lymphocyte proliferation were obtained on isolated T lymphocytes rather than specific Th subpopulations. This provides a global overview of these functions and is a limitation of our study. However, pro-inflammatory T cell populations, such as Th1, Th2, Th17, and Treg, can be present in relatively small numbers, making an accurate analysis challenging. Studies have demonstrated that the isolation and characterization of these specific cell subsets often result in limited cell viability and reduced functional yields.

A more in-depth analysis of the signaling pathways, particularly PPAR, is needed to fully understand their contribution to these observed effects. The analysis of specific markers, such as CD40L and CD25, was also limited because of a few options of antibodies that detect rat proteins for a flow cytometry analysis. CD40 is a co-stimulatory protein found on the surface of various immune cells, including B cells, dendritic cells, and macrophages. It plays a pivotal role in immune regulation by facilitating interactions between T cells and antigen-presenting cells (APCs). When CD40 binds to its ligand (CD40L), primarily expressed on activated T helper cells [82], it triggers a cascade of intracellular signaling pathways that enhance cell activation, proliferation, and survival. CD25, on the other hand, is expressed on activated T cells, particularly Tregs [83]. CD25 plays a significant role in T cell activation and proliferation by mediating the effects of IL-2, a critical growth factor for T cells. In the context of the observed effects, the presence of CD25 can indicate T cell activation and the potential regulatory mechanisms in play, highlighting the balance between immune activation and regulation. Including these markers in future studies could offer further insights into the immune modulation promoted by ω-3 fatty acids.

In addition, for future studies, exploring nanocarrier technologies, such as liposomal encapsulation, is recommended to improve the bioavailability and stability of ω-3 fatty acids. These technologies protect compounds from oxidation and degradation during storage and metabolism, ensuring greater efficacy in delivering ω-3 to the body [84]. Additionally, Rasti et al. [85] have reported that liposome and nanoliposome systems exhibit superior oxidative and physical stability, which underscores their potential in food supplementation applications and nutritional therapies. Including these approaches in future experimental protocols may provide valuable insights into the impact of nanoencapsulation on the absorption and therapeutic effects of ω-3 fatty acids, enhancing the benefits observed with traditional supplementation methods.

## 5. Conclusions

Our findings on the supplementation of a nonobese T2DM model with FO rich in ω-3 fatty acids highlighted its positive influence on glycemic, insulin, and lipid parameters. The analysis of the CD4+ T lymphocyte profile revealed a shift from a pro-inflammatory to a regulatory profile in GK rats treated with ω-3 fatty acids, with an increase in CD4+ Treg lymphocytes and a reduction in Th1 and Th17 profiles. This enhanced regulatory immune response was supported by a gene expression analysis, demonstrating the modulation of genes associated with Treg lymphocytes. The complex interaction between ω-3 fatty acids and T lymphocyte metabolism, including the reduction in GLUT-1 expression and glucose uptake, underscores the potential of ω-3 fatty acids in the management of T2DM, offering both metabolic and immunological therapeutic perspectives for nonobese patients with T2DM.

## Figures and Tables

**Table 1:** Sequences of the primers used for the RT-PCR experiments.

Gene	Forward Sequence	Reverse Sequence
Rplp0	GAACATCTCCCCCTTCTCCTCC	ATTGCGGACACCCTCTAGGAA
T-bet	GAGCCCCACGAGCAATTACAG	GTATAAGCGGTTCCCTGGCA
IFN-γ	TGTCATCGAATCGCACCTGA	TGTGGGTTGTTCACCTCGAA
TNF-α	ATGGGCTCCCTCTCATCAGT	GTCTGGTGGTTTGCTACGAC
IL-18	GACCGAACAGCCAACGAATC	GATAGGGTCACAGCCAGTCC
IL-2	CTGCAGCGTGTGTTGGATT	GGCTCATCATCGAATTGGCAC
GATA-3	AGTACCCCCTGACGGAAGAG	TAGTAGGACGGGAGTGGTT
IL-4	GTACCGGGAACGGTATCCAC	TTCTCCGTGGTGTTGACCTG
RORα	AACATCTCGGGAGTTGCTGG	AGGAGTAGGCCACATTGCAC
TGF-α	CTGCTGACCCCCACTGATA	AGCCCTGTATTCCGTCTCCT
IL-17	GGAGAATTCCATCCATGTGCC	ATGAGTACCGCTGCCTTCAC
IL-6	ACAAGTCCGGAGAGGAGACT	GAATTGCCATTGACAAACTCT
FOXP3	ACTCTGCCTTCACACGAGAC	GAGGCAGGCTGGATAACGG
IL-35	GTCTCTGGACCTGCCAAGTG	CCAGTGTGCTGGTTTTGTCC
IL-10	AAGACCCAGACATCAAGGCG	AATCGATGACAGCGCCGTAG
Glut-1	GTCGTACGGCAAGATCGCTGAG	TTCAATCATGTCACCCACGCTG

## Data Availability

The data that support the findings of this study are available in the Supplementary Materials.

## Supplementary Materials

The following supporting information can be downloaded at: https://www.mdpi.com/article/10.3390/nu16234106/s1. The Supplementary Materials contain the individual data for body weight (Table S1), adiposity (Table S2), daily energy consumption (Table S3), GTT (Table S4), ITT (Table S5), fasting glucose (Table S6), insulin concentration (Table S7), serum biochemical parameters (Table S8), 2-NBDG uptake (Table S9), percentage of Th1 cells (Table S10), percentage of Th2 cells (Table S11), percentage of Th17 cells (Table S12), percentage of Treg (Table S13), proliferative capacity (Table S14), cytokine concentration (Table S15) and gene expression (Table S16).
