# Supplementary material for: Omega-3 Fatty Acids Weaken Lymphocyte Inflammatory Features and Improve Glycemic Control in Nonobese Diabetic Goto-Kakizaki Rats
Source: Nutrients. 2024 Nov 28;16(23):4106. doi: 10.3390/nu16234106 (PMC11644024; doi:10.3390/nu16234106)
Supplement: Supplementary file 1 [file nutrients-16-04106-s001.zip › nutrients-3279546-supplementary.pdf]

SUPPLEMENTAL DATA

S1: Gating Strategy: Lymphocyte populations were identified in gate P1 (A), with doublet exclusion performed in gate P2 (B). Panel (C) shows the analysis of cells positive for CD4-FITC (FL1) and TNF-α-Alexa 647 (FL4). Panel (D) represents the negative control. The subsequent panels display the positive controls for CD4 (E), TNF-α (F), IL-4 (G), ROR-γ (H), and FOXP3 (I).

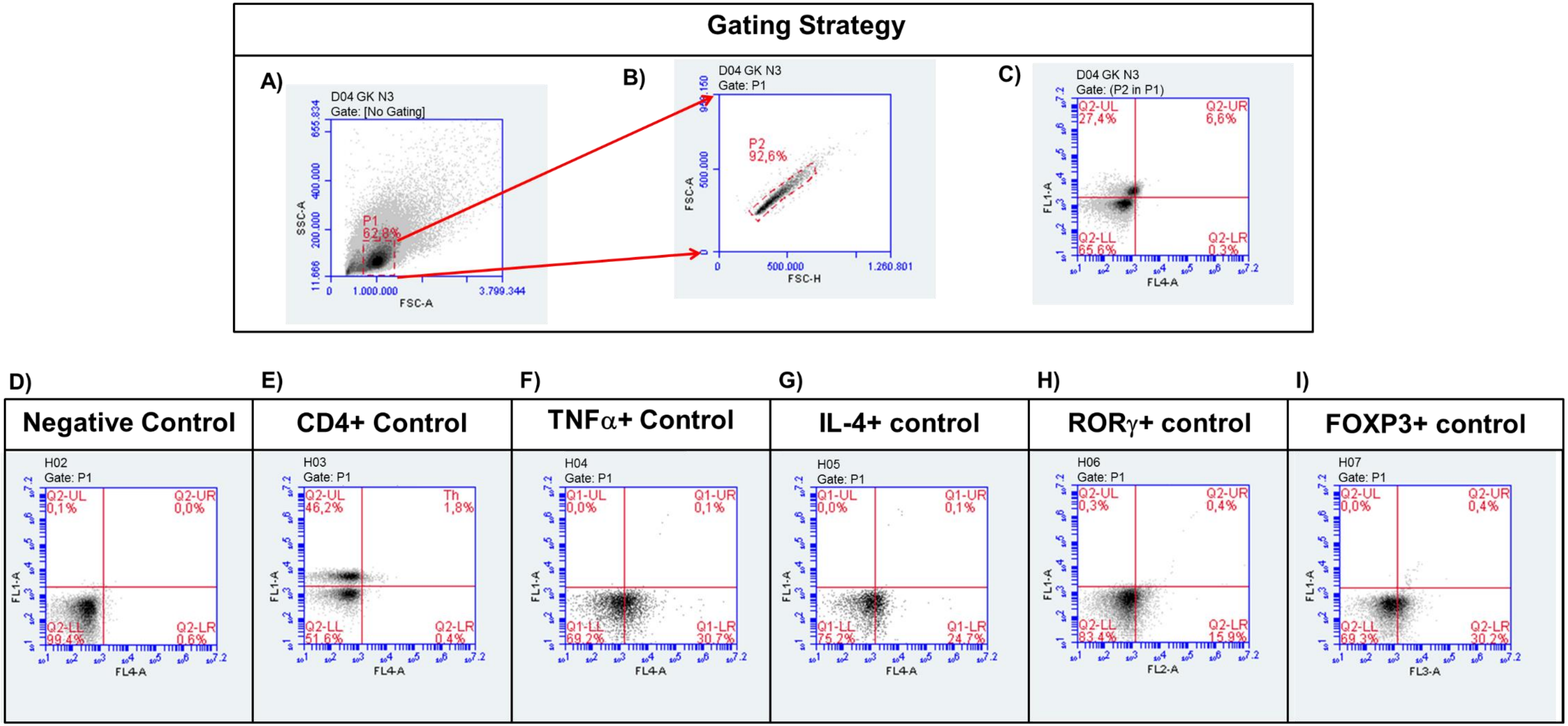

S2: On the left, representative flow cytometry dot plots for each cell profile are shown, while on the right, the mean intracellular fluorescence of cytokines from each CD4 positive profile is presented.

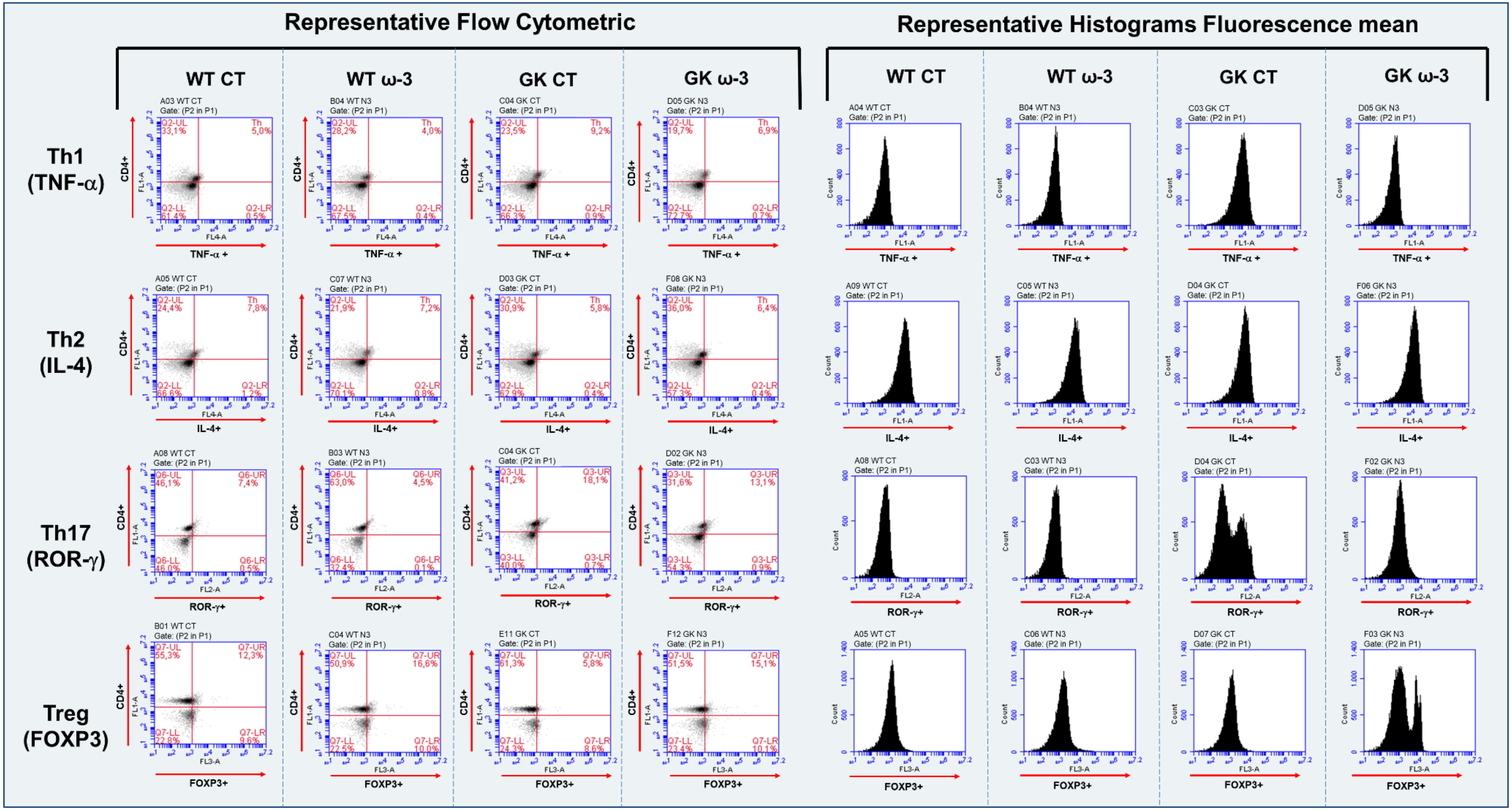

S3: Representative histograms of BrdU incorporation, CD28 expression, and 2-NBDG uptake are presented below.

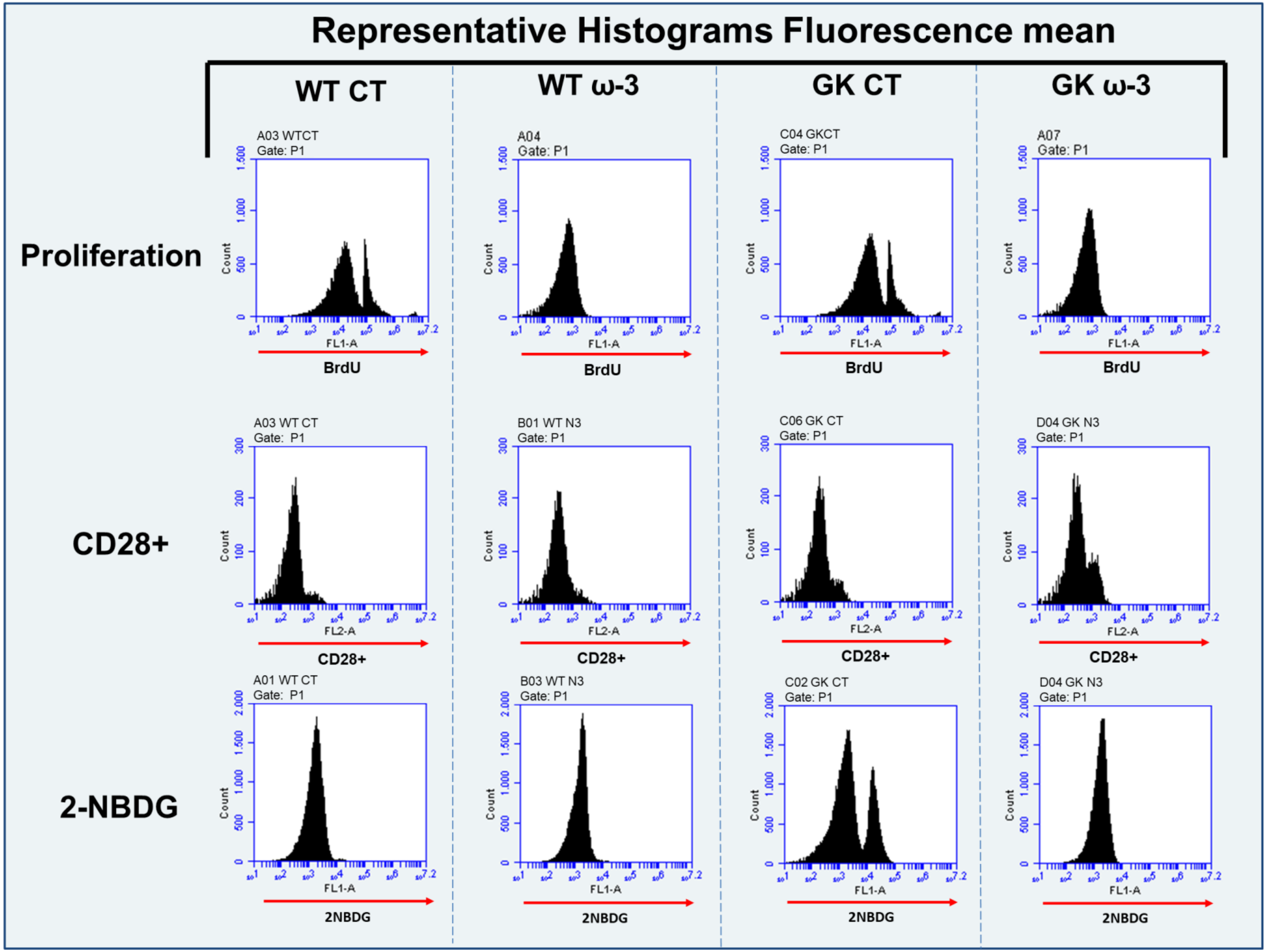

Table S1. Weekly control of the body weight (g) of WT CT (n=15), WT ω-3 (n=11), GK CT (n=14), and GK ω-3 (n=15) rats. WT, Wistar; CT, control; GK, Goto-Kakizaki.

	Weekly evolution of body mass (g)														
	Animal number														
	1	2	3	4	5	6	7	8	9	10	11	12	13	14	15
Week	WT CT														
1	307	290	310	308	300	340	300	300	302	276	277	206	320	350	355
2	332	318	345	328	327	359	318	328	337	300	301	304	340	337	372
3	318	329	363	366	381	380	320	360	360	322	326	314	357	368	376
4	338	339	375	377	388	384	334	380	368	337	340	333	377	400	400
5	338	339	375	377	388	384	334	397	392	357	359	327	393	424	412
6	382	340	395	389	363	427	368	415	395	364	381	349	410	445	442
7	407	373	417	398	375	439	382	436	406	380	378	359	410	469	442
8	421	385	422	402	382	456	386	447	420	497	395	371	434	368	463
Week	WT ω-3														
1	300	305	297	315	337	347	300	390	329	267	284	-	-	-	-
2	315	337	272	342	364	360	310	350	339	251	275	-	-	-	-
3	337	359	294	340	380	377	324	364	347	365	282	-	-	-	-
4	350	380	298	348	385	376	330	359	345	380	299	-	-	-	-
5	350	380	298	348	385	376	351	373	389	362	321	-	-	-	-
6	361	401	318	373	439	403	413	452	428	350	370	-	-	-	-
7	393	440	348	415	458	428	433	353	401	374	394	-	-	-	-
8	397	448	442	428	476	430	439	479	459	385	401	-	-	-	-
Week	GK CT														
1	314	304	300	284	287	280	288	255	275	269	250	265	239	250	-
2	325	309	310	292	301	297	297	270	280	280	258	278	274	270	-
3	335	311	320	312	312	308	311	279	299	301	272	291	290	287	-
4	355	335	338	323	329	323	325	293	318	314	285	304	306	301	-
5	360	341	345	328	330	340	330	302	334	329	299	327	316	315	-
6	365	354	363	335	334	349	345	312	330	334	313	340	320	325	-
7	383	369	375	354	355	363	365	314	350	353	322	350	329	333	-
8	390	384	383	358	362	366	371	317	350	362	325	364	335	338	-
Week	GK ω-3														
1	260	259	280	285	272	285	311	266	279	275	266	261	269	279	275
2	291	278	297	293	298	305	321	259	271	271	270	267	265	280	270
3	318	298	300	300	292	307	337	258	261	259	264	260	260	278	273
4	327	307	303	321	311	316	347	270	274	282	289	283	281	296	298
5	328	317	314	325	318	319	350	297	304	303	311	301	304	322	320
6	332	327	331	339	326	315	361	314	320	309	308	318	315	330	336
7	339	342	335	338	349	339	378	324	326	325	328	328	321	340	343
8	350	345	346	320	350	338	378	342	341	346	336	342	334	360	351

Table S2. Adiposity was determined based on the sum of the retroperitoneal, renal, epididymal, and subcutaneous adipose tissues. WT CT (n=15), WT ω-3 (n=11), GK CT (n=14), and GK ω-3 (n=15) rats. WT, Wistar; CT, control; GK, Goto-Kakizaki.

Adiposity (g)				
Animal number	WT CT	WT ω-3	GK CT	GK ω-3
1	3.92	6.67	6.07	4.54
2	5.15	8.91	4.71	4.70
3	7.98	5.39	6.69	1.61
4	4.72	8.95	4.27	4.78
5	7.09	12.05	4.88	4.42
6	11.24	8.11	7.62	4.89
7	5.28	8.20	6.50	6.06
8	7.17	8.24	4.82	5.85
9	8.22	10.99	5.92	5.61
10	10.37	9.41	5.13	4.63
11	8.07	8.71	7.03	5.86
12	11.23	-	5.86	2.69
13	12.40	-	6.73	6.74
14	13.36	-	6.79	5.03
15	9.00	-	-	5.45
Mean (g)	8.34	8.69	5.93	4.85
Standard deviation (SD)	2.90	1.81	1.02	1.29

Table S3. Daily energy consumption (kcal) of the experimental groups of rats: WT CT (n=15), WT ω-3 (n=11), GK CT (n=14), and GK ω-3 (n=15), calculated based on food intake and the calorific value of the Nuvilab CR-1 diet. WT, Wistar; CT, control; GK, Goto-Kakizaki.

	**Energy consumption (kcal/day)**			
Animal number	WT CT	WT ω-3	GK CT	GK ω-3
1	76.20	65.60	51.10	52.10
2	75.40	58.80	57.40	36.50
3	78.70	59.00	52.50	43.60
4	74.80	57.20	54.90	47.60
5	73.90	59.20	51.10	48.30
6	79.10	66.90	52.10	49.70
7	79.70	67.90	50.20	53.30
8	75.30	66.90	52.20	50.40
9	76.00	55.30	53.20	51.10
10	70.10	64.00	42.00	38.00
11	65.10	64.50	42.40	41.40
12	71.60	-	46.70	40.90
13	70.60	-	45.00	42.20
14	70.40	-	43.30	41.10
15	70.70	-	-	31.70
Mean (kcal/day)	73.84	62.30	49.58	44.53
Standard deviation (SD)	4.04	4.47	4.86	6.40

Table S4. Glucose tolerance test (GTT, mg/dL) was performed with glucose measurements at 0 min (before injection) and at 15, 30, and 60 min after intraperitoneal administration of 50% glucose solution (2 g/kg) in WT CT (n=15), WT ω-3 (n=11), GK CT (n=14), and GK ω-3 (n=15) rats. WT, Wistar; CT, control; GK, Goto-Kakizaki.

Glucose tolerance test (GTT)																
Animal number		1	2	3	4	5	6	7	8	9	10	11	12	13	14	15
Group	Time (min)	Glucose concentration (mg/dL)														
WT CT	0	99	103	105	102	104	112	103	94	93	86	85	81	81	91	88
	15	129	116	237	233	249	118	138	207	197	173	209	206	201	190	218
	30	125	130	226	180	212	165	131	145	160	170	145	190	118	174	162
	60	132	115	155	135	148	121	129	115	122	128	105	111	106	115	124
WT ω-3	0	92	91	108	103	96	116	85	92	84	83	91	-	-	-	-
	15	207	266	127	230	201	178	191	180	242	166	175	-	-	-	-
	30	230	239	122	173	240	175	151	185	134	144	202	-	-	-	-
	60	131	166	111	135	153	154	93	108	119	119	126	-	-	-	-
GK CT	0	183	198	237	185	192	197	190	216	224	213	228	230	240	199	-
	15	240	288	353	354	304	473	237	300	373	324	380	399	391	368	-
	30	272	258	382	451	296	553	282	489	388	373	382	390	384	380	-
	60	369	305	402	488	297	475	341	397	387	374	360	354	419	410	-
GK ω-3	0	105	133	135	140	176	130	118	145	136	134	133	132	140	142	140
	15	189	256	282	254	296	200	302	234	197	214	251	285	238	217	247
	30	332	320	230	359	340	256	232	190	199	288	282	295	201	237	247
	60	284	300	207	290	278	260	264	180	178	166	181	184	191	183	172

Table S5. Insulin tolerance test (ITT, mg/dL) was performed with glucose measurements at 0 min (before injection) and at 4, 8, 12, and 20 min after intraperitoneal administration of insulin (0.75 IU/kg) in WT CT (n=15), WT ω-3 (n=11), GK CT (n=14), and GK ω-3 (n=15) rats. WT, Wistar; CT, control; GK, Goto-Kakizaki.

Insulin tolerance test (ITT)																
Animal number		1	2	3	4	5	6	7	8	9	10	11	12	13	14	15
Group	Time (min)	Glucose concentration (mg/dL)														
WT CT	0	112	126	101	98	91	96	107	105	95	85	80	82	79	97	97
	4	115	133	125	104	101	110	111	130	93	107	84	95	118	93	100
	8	100	132	144	149	106	121	96	114	88	86	97	98	104	111	90
	12	103	108	120	93	108	89	101	87	68	63	98	68	95	80	71
	20	93	100	71	88	90	85	95	74	58	68	70	65	87	68	65
WT ω-3	0	108	125	121	99	106	91	99	104	83	82	109	-	-	-	-
	4	108	122	116	101	126	119	121	89	107	93	94	-	-	-	-
	8	102	116	112	127	123	117	82	69	100	110	108	-	-	-	-
	12	95	90	81	76	104	86	90	72	88	121	95	-	-	-	-
	20	78	84	86	72	90	89	90	68	90	101	90	-	-	-	-
GK CT	0	210	255	233	231	176	152	187	167	220	189	190	182	197	183	-
	4	300	290	267	206	212	280	260	177	251	180	207	162	214	172	-
	8	280	293	240	220	205	265	247	181	245	242	191	172	207	201	-
	12	284	229	267	218	292	259	210	166	186	138	198	151	137	167	-
	20	241	175	230	184	253	221	189	139	172	121	115	114	132	116	-
GK ω-3	0	115	130	142	105	124	126	125	120	132	123	101	128	104	136	123
	4	273	172	277	278	226	210	225	257	182	163	140	174	220	164	154
	8	168	154	180	181	124	193	189	170	230	138	116	183	174	175	168
	12	160	141	171	178	129	106	102	126	182	137	119	71	63	104	139
	20	123	133	148	139	124	131	128	137	209	118	69	118	104	94	96

Table S6. Fasting glucose measurements were taken in WT CT (n=6), WT ω-3 (n=6), GK CT (n=6), and GK ω-3 (n=6) rats. WT, Wistar; CT, control; GK, Goto-Kakizaki.

**Fasting glucose (mg/dL)**				
Group	WT CT	WT ω-3	GK CT	GK ω-3
Animal	Glucose (mg/dL)	Glucose (mg/dL)	Glucose (mg/dL)	Glucose (mg/dL)
1	128.60	164.70	359.30	135.50
2	187.20	109.60	315.30	251.60
3	170.20	101.90	423.30	247.50
4	128.40	135.90	312.20	194.70
5	170.10	100.10	300.10	201.40
6	129.10	130.90	390.50	187.80
Mean (mg/dL)	152.30	123.90	350.10	203.10
Standard deviation (SD)	26.56	24.95	49.44	42.90

Table S7. Insulin measurements (ng/mL) were taken in WT CT (n=6), WT ω-3 (n=6), GK CT (n=7), and GK ω-3 (n=7) rats. WT, Wistar; CT, control; GK, Goto-Kakizaki.

**Insulin concentration (ng/mL)**				
Group	WT CT	WT ω-3	GK CT	GK ω-3
Animal	Insulin (ng/mL)	Insulin (ng/mL)	Insulin (ng/mL)	Insulin (ng/mL)
1	0.42	0.37	0.56	0.37
2	0.30	0.35	0.83	0.58
3	0.27	0.49	0.60	0.26
4	0.66	0.48	0.98	0.50
5	1.31	0.30	0.98	0.60
6	0.25	0.56	1.11	0.77
7	-	-	0.81	0.74
Mean (ng/mL)	0.54	0.42	0.84	0.55
Standard deviation (SD)	0.40	0.10	0.20	0.18

Table S8. Fasting serum biochemical parameters, including creatine kinase (U/L), lactate dehydrogenase (U/L), C-reactive protein (mg/dL), total cholesterol (mg/dL), and low-density lipoprotein (mg/dL), were measured in WT CT (n=6), WT ω-3 (n=6), GK CT (n=6), and GK ω-3 (n=6) rats. Triglycerides (mg/dL) were assessed in the same groups, with n=10 for each. WT, Wistar; CT, control; GK, Goto-Kakizaki.

Animal	Creatine Kinase (U/L)	Lactate dehydrogenase (U/L)	C-Reactive protein (mg/dL)	Total cholesterol (mg/dL)	Triglycerides (mg/dL)	Low-density lipoprotein (mg/dL)
WT CT	3.90	157.00	2.62	66.00	226.34	38.30
	5.30	412.00	1.84	66.60	131.49	38.90
	4.20	376.00	1.68	67.20	140.76	39.40
	4.50	385.00	1.36	66.50	158.61	38.80
	4.20	276.00	1.68	65.10	167.26	37.40
	4.50	185.00	1.36	67.60	137.49	39.80
	-	-	-	-	136.94	-
	-	-	-	-	135.85	-
	-	-	-	-	175.99	-
	-	-	-	-	167.73	-
Mean	4.43	298.50	1.75	66.50	157.80	38.77
Standard deviation (SD)	0.48	109.30	0.46	0.88	28.92	0.84
WT ω-3	4.60	63.00	2.26	63.00	100.94	30.90
	5.40	162.00	1.06	64.60	122.99	32.50
	3.00	168.00	1.41	64.10	126.27	32.00
	5.20	106.00	1.39	64.50	120.73	32.40
	4.00	168.00	1.41	64.10	121.67	32.00
	5.20	106.00	1.39	64.70	106.39	32.60
	-	-	-	-	117.07	-
	-	-	-	-	130.71	-
	-	-	-	-	131.72	-
	-	-	-	-	123.38	-
Mean	4.56	128.80	1.48	64.17	120.20	32.07
Standard deviation (SD)	0.92	43.69	0.40	0.62	9.84	0.62

Animal	Creatine Kinase (U/L)	Lactate dehydrogenase (U/L)	C-Reactive protein (mg/dL)	Total cholesterol (mg/dL)	Triglycerides (mg/dL)	Low-density lipoprotein (mg/dL)
GK CT	4.80	993.00	4.38	89.40	186.59	51.90
	6.60	491.00	6.09	90.50	135.85	53.00
	4.20	548.30	5.55	90.30	177.55	52.80
	6.40	545.00	5.06	87.40	141.70	49.90
	7.20	818.30	4.55	87.30	190.65	49.80
	6.40	345.10	5.06	89.20	83.16	51.70
	-	-	-	-	322.68	-
	-	-	-	-	211.15	-
	-	-	-	-	180.05	-
	-	-	-	-	96.02	-
Mean	5.93	623.50	5.11	89.02	172.50	51.52
Standard deviation (SD)	1.16	237.20	0.63	1.38	67.31	1.38
GK ω-3	3.40	495.90	2.19	70.40	110.68	36.70
	4.60	353.50	2.28	69.80	131.41	36.20
	5.70	360.30	3.83	70.20	108.18	36.50
	4.90	355.00	6.87	72.80	167.89	39.10
	5.70	463.00	4.83	70.90	110.91	37.30
	4.90	455.00	1.87	70.30	106.47	36.60
	-	-	-	-	146.53	-
	-	-	-	-	93.45	-
	-	-	-	-	88.23	-
	-	-	-	-	82.62	-
Mean	4.86	413.80	3.64	70.73	114.60	37.07
Standard deviation (SD)	0.85	64.52	1.94	1.07	26.76	1.06

Table S9. 2-NBDG uptake in lymphocytes stimulated with PMA and ionomycin was measured in WT CT (n=5), WT ω-3 (n=5), GK CT (n=5), and GK ω-3 (n=5) rats. WT, Wistar; CT, control; GK, Goto-Kakizaki; PMA, phorbol myristate acetate.

**2-NBDG Uptake (fluorescence mean intensity)**				
Animal number	WT CT	WT ω-3	GK CT	GK ω-3
1	5,05	4,053	8,943	4,029
2	6,005	5,674	7,493	6,937
3	7,004	6,094	9,387	6,743
4	6,584	5,693	7,365	5,839
5	5,987	4,983	8,473	5,002
Mean	6,126	5,299	8,332	5,71
Standard deviation (SD)	737	803	887	1,216

Table S10. Percentage of CD4+TNF-α+ cells and expression of TNF-α (fluorescence mean) in lymphocytes stimulated with PMA and ionomycin. WT CT (n=8), WT ω-3 (n=8), GK CT (n=8), and GK ω-3 (n=8) rats. WT, Wistar; CT, control; GK, Goto-Kakizaki; PMA, phorbol myristate acetate.

**Th1 Profile**								
	% Total lymphocyte ($CD4^{+}$-TNF-α^{+})				Fluorescence mean (TNF-α)			
	WT CT	WT ω-3	GK CT	GK ω-3	WT CT	WT ω-3	GK CT	GK ω-3
	5.68	4.54	9.74	5.26	1,31	1,08	5,35	1,32
	6.41	4.68	7.47	5.14	1,55	2,00	4,18	2,35
	5.00	4.10	8.04	6.61	1,82	1,03	5,52	1,60
	5.87	4.00	9.20	6.61	1,38	2,08	5,48	1,72
	6.62	3.67	8.92	6.90	1,01	1,08	4,26	2,55
	4.08	6.56	8.64	6.58	1,21	1,48	4,85	1,94
	6.81	3.86	10.39	7.51	1,09	1,06	4,99	2,37
	4.82	5.19	11.56	6.95	1,12	1,79	6,55	2,46
Mean	5.66	4.58	9.25	6.45	1,31	1,45	5,15	2,04
Standard deviation (SD)	0.96	0.94	1.30	0.82	2.70	4.49	7.66	4.56

Table S11. Percentage of CD4+IL4+ cells and intracellular expression of IL-4 (fluorescence mean) in lymphocytes stimulated with PMA and ionomycin were measured in WT CT (n=13), WT ω-3 (n=11), GK CT (n=13), and GK ω-3 (n=13) rats. WT, Wistar; CT, control; GK, Goto-Kakizaki; PMA, phorbol myristate acetate.

**Th2 Profile**								
	% Total lymphocyte ($CD4^{+}$-IL-4^{+})				Fluorescence mean (IL-4)			
	WT CT	WT ω-3	GK CT	GK ω-3	WT CT	WT ω-3	GK CT	GK ω-3
	5.65	9.17	6.27	5.86	1,21	2,32	1,26	1,37
	7.55	6.92	6.00	5.24	1,45	2,20	1,80	1,41
	7.11	6.04	5.85	5.40	1,15	2,07	1,87	1,67
	7.49	7.76	5.43	7.05	1,78	1,77	1,10	1,01
	7.80	7.94	5.39	6.94	1,01	1,18	1,18	1,00
	6.18	7.16	5.26	5.35	1,45	1,57	1,79	1,02
	6.87	7.20	5.93	7.30	1,63	2,00	1,05	1,16
	6.56	5.69	6.25	6.40	1,59	1,27	1,49	1,53
	9.01	9.49	7.25	5.85	1,63	1,20	1,21	1,15
	5.25	7.40	7.84	9.29	1,41	1,38	1,03	1,74
	5.56	7.58	4.22	9.59	1,93	2,05	1,50	1,68
	8.16	-	4.70	9.81	1,81	-	1,04	1,87
	9.26	-	6.12	5.75	1,97	-	1,86	1,24
Mean	7.11	7.49	5.89	6.91	1,54	1,73	1,40	1,37
Standard deviation (SD)	1.27	1.13	0.95	1.65	2.97	4.25	3.34	3.02

Table S12. Percentage of CD4+ROR-γ+ cells and intracellular expression of ROR-γ (fluorescence mean) in lymphocytes stimulated with PMA and ionomycin were measured in WT CT (n=8), WT ω-3 (n=8), GK CT (n=8), and GK ω-3 (n=8) rats. WT, Wistar; CT, control; GK, Goto-Kakizaki; PMA, phorbol myristate acetate.

**Th17 Profile**								
	% Total lymphocyte ($CD4^+$-ROR-γ^+)				Fluorescence mean (ROR-γ)			
	WT CT	WT ω-3	GK CT	GK ω-3	WT CT	WT ω-3	GK CT	GK ω-3
	5.89	5.90	19.39	14.74	2,15	2,16	5,17	2,99
	8.62	5.59	19.93	13.10	2,22	2,70	5,75	2,92
	4.32	4.53	16.86	11.15	2,17	2,41	4,40	3,20
	8.56	4.80	18.16	16.41	2,19	2,52	4,43	2,60
	6.63	3.92	21.04	14.40	2,82	2,55	3,07	3,31
	9.57	5.16	15.90	11.80	2,07	2,04	3,40	2,92
	5.40	4.36	18.97	11.77	2,60	2,30	5,48	3,56
	7.49	2.39	18.75	11.87	2,92	2,20	3,82	3,42
Mean	7.06	4.58	18.63	13.16	2,39	2,36	4,44	3,12
Standard deviation (SD)	1.81	1.09	1.65	1.85	3.34	2.23	9.76	3.14

Table S13. Percentage of CD4+FOXP3+ cells and intracellular expression of FOXP3 (fluorescence mean) in lymphocytes stimulated with PMA and ionomycin were measured in WT CT (n=13), WT ω-3 (n11), GK CT (n=13, and GK ω-3 (n=13) rats. WT, Wistar; CT, control; GK, Goto-Kakizaki; PMA, phorbol myristate acetate.

**Treg Profile**								
	% Total lymphocyte ($CD4^+$ $FOXP3^+$)				Fluorescence mean (FOXP3)			
	WT CT	WT ω-3	GK CT	GK ω-3	WT CT	WT ω-3	GK CT	GK ω-3
	11.92	14.22	2.52	11.91	3,43	4,40	2,73	3,09
	11.44	15.84	5.22	10.37	3,12	3,98	2,85	3,54
	15.32	16.22	7.08	12.04	3,01	3,81	3,49	4,15
	11.63	16.64	4.88	13.86	2,87	3,83	3,25	4,70
	11.70	17.93	3.04	12.42	3,04	3,31	3,31	3,95
	12.72	16.99	6.31	15.97	3,48	3,92	3,08	4,86
	15.84	18.96	5.32	16.92	3,27	3,98	2,95	4,24
	10.04	17.35	2.75	17.85	3,78	3,80	3,42	4,13
	7.34	16.90	4.72	17.23	3,13	3,26	3,83	4,56
	11.71	18.74	4.16	19.50	3,74	2,18	2,48	4,44
	14.24	15.78	5.80	17.01	2,58	2,39	3,36	4,28
	15.13	-	10.31	15.10	3,41	-	4,41	3,50
	12.30	-	13.04	18.68	3,89	-	3,82	3,78
Mean	12.41	16.87	5.78	15.30	3,29	3,53	3,31	4,09
Standard deviation (SD)	2.33	1.37	2.99	2.91	3.80	6.91	5.17	5.12

Table S14. Proliferative capacity of lymphocytes stimulated with concanavalin A (5 µg/mL) and incubated with 5 µM BrdU for 48 hours, along with CD28+ expression, was measured in WT CT (n=8), WT ω-3 (n=8), and GK CT (n=8) rats. WT, Wistar; CT, control; GK, Goto-Kakizaki.

	**Fluorescence mean**							
	**Proliferation assay**				**CD28^{+} assay**			
	WT CT	WT ω-3	GK CT	GK ω-3	WT CT	WT ω-3	GK CT	GK ω-3
	65,595	49,011	69,844	48,914	2,042	2,091	1,875	2,052
	72,126	57,230	66,150	59,878	2,044	2,079	1,871	1,949
	80,897	50,345	79,855	42,628	1,797	1,756	1,690	1,842
	78,417	62,487	80,497	61,343	1,760	1,727	1,609	1,784
	81,749	50,867	77,737	53,584	1,725	1,710	2,241	1,718
	80,123	50,463	82,036	56,148	2,349	2,287	2,301	2,247
	76,532	56,369	87,536	64,529	2,720	2,383	2,242	2,277
	78,166	65,987	75,963	47,330	2,267	2,079	-	2,516
Mean	76,701	55,345	77,452	54,294	2,088	2,014	1,975	2,048
Standard deviation (SD)	5,401	6,300	6,821	7,589	344	258	284	278

Table S15. Cytokine concentrations (pg/mL) in the culture supernatant of lymphocytes stimulated with PMA and ionomycin, including IL-10, IFN-γ, TNF-α, IL-17F, and IL-17A, were measured in WT CT (n=6), WT ω-3 (n=6), GK CT (n=6), and GK ω-3 (n=6) rats. WT, Wistar; CT, control; GK, Goto-Kakizaki.

Concentration of cytokines (pg/mL)					
Cytokine	IL-10	IFN-γ	TNF-α	IL-17 F	IL-17 A
WT CT	1.22	5.09	5.75	1.22	7.30
	8.23	6.42	6.26	6.02	7.23
	6.54	1.22	7.32	3.41	4.42
	6.42	5.33	6.79	5.21	3.45
	1.22	1.22	4.56	1.22	4.30
	1.22	3.63	5.78	6.63	7.00
Mean (pg/mL)	4.14	3.82	6.08	3.95	5.62
Standard deviation (SD)	3.26	2.20	0.96	2.38	1.74
WT ω-3	9.82	5.30	7.89	2.88	2.54
	1.22	4.31	2.03	6.94	3.81
	6.77	5.35	1.22	1.22	1.22
	12.41	4.62	6.50	4.06	5.73
	7.85	2.72	5.60	2.53	3.30
	12.65	4.47	7.67	3.47	3.02
Mean (pg/mL)	8.45	4.46	5.15	3.52	3.27
Standard deviation (SD)	4.26	0.96	2.87	1.93	1.49
GK CT	1.22	15.46	17.53	8.41	14.50
	1.22	14.70	14.22	5.82	13.51
	6.07	11.22	14.48	1.22	14.20
	5.66	16.05	16.63	9.42	13.39
	4.00	14.93	14.03	9.71	18.93
	3.12	14.37	15.71	8.91	12.90
Mean (pg/mL)	3.55	14.46	15.43	7.25	14.57
Standard deviation (SD)	2.10	1.69	1.43	3.26	2.21
GK ω-3	11.22	2.74	5.31	4.33	4.13
	17.13	1.22	9.33	1.22	4.41
	12.61	5.24	6.09	3.13	6.48
	15.07	6.18	7.04	1.22	5.32
	11.00	4.22	3.17	5.12	5.59
	24.00	6.85	8.88	3.34	6.52
Mean (pg/mL)	15.17	4.41	6.64	3.06	5.41
Standard deviation (SD)	4.93	2.13	2.30	1.59	1.01

Table S16. Expression of genes (arbitrary units) related to different lymphocyte profiles and glucose transporter 1 (GLUT1), with RPLP0 as the constitutive gene. Outliers were excluded during statistical analysis by Rout test (*). WT, Wistar; CT, control; GK, Goto-Kakizaki.

- **Th1 Profile:**
  - T-bet: WT CT (n=15), WT ω-3 (n=11), GK CT (n=14), GK ω-3 (n=13)
  - IFN-γ: WT CT (n=15), WT ω-3 (n=9), GK CT (n=13), GK ω-3 (n=13)
  - TNF-α: WT CT (n=15), WT ω-3 (n=11), GK CT (n=13), GK ω-3 (n=14)
  - IL-18: WT CT (n=15), WT ω-3 (n=11), GK CT (n=13), GK ω-3 (n=14)
  - IL-2: WT CT (n=12), WT ω-3 (n=11), GK CT (n=11), GK ω-3 (n=15)
- **Th2 Profile:**
  - GATA-3: WT CT (n=14), WT ω-3 (n=11), GK CT (n=12), GK ω-3 (n=15)
  - IL-4: WT CT (n=14), WT ω-3 (n=10), GK CT (n=14), GK ω-3 (n=13)
- **Th17 Profile:**
  - ROR-γ: WT CT (n=15), WT ω-3 (n=11), GK CT (n=12), GK ω-3 (n=15)
  - IL-17: WT CT (n=15), WT ω-3 (n=11), GK CT (n=14), GK ω-3 (n=13)
  - IL-6: WT CT (n=14), WT ω-3 (n=10), GK CT (n=14), GK ω-3 (n=14)
  - TGF-β: WT CT (n=15), WT ω-3 (n=11), GK CT (n=12), GK ω-3 (n=15)
- **Treg Profile:**
  - FOXP3: WT CT (n=11), WT ω-3 (n=8), GK CT (n=13), GK ω-3 (n=15)
  - IL-10: WT CT (n=13), WT ω-3 (n=8), GK CT (n=13), GK ω-3 (n=13)
  - IL-35: WT CT (n=14), WT ω-3 (n=11), GK CT (n=14), GK ω-3 (n=15)
- **GLUT1:**
  - WT CT (n=15), WT ω-3 (n=11), GK CT (n=14), GK ω-3 (n=15)

Group	T-bet	IFN-γ	TNF-α	IL-18	IL-2	GATA-3	IL-4	ROR-γ	IL-17	IL-6	TGF-β	FOXP3	IL-10	IL-35	GLUT-1
	**Gene expression ($2^{-delta\ delta\ CT}$)**														
	**Arbitrary units (AU)**														
WT CT	1.81	3.49	1.16	1.67	1.39	0.76	1.14	2.36	1.84	1.38	1.45	1.25	0.91	2.17	0.38
	1.64	1.90	0.94	0.49	0.08	0.63	1.60	2.13	2.38	0.75	1.22	0.78	1.05	3.38	0.90
	2.49	2.39	1.01	0.50	0.34	0.96	1.21	1.82	2.74	0.53	1.49	1.04	1.29	5.84*	0.94
	0.27	4.15	0.73	1.77	0.45	0.80	0.14	1.61	0.46	1.49	1.42	1.12	0.67	1.88	1.51
	1.90	3.22	1.21	2.82	1.26	1.11	0.63	3.02	1.59	0.61	1.71	2.01	1.65	2.47	3.72
	2.30	2.66	1.44	0.60	0.31	1.17	0.27	0.76	1.68	0.83	1.15	1.20	1.00	1.03	1.32
	3.27	5.57	2.34	1.21	0.07	0.46	1.53	0.84	1.41	0.71	1.00	0.46	0.90	0.72	2.91
	2.19	0.96	0.57	1.47	0.71	2.09	1.82	1.51	0.93	1.62	0.64	2.46	4.47*	0.45	1.96
	0.24	0.16	0.39	0.23	0.53	0.50	0.48	0.40	0.34	2.64	0.44	5.38*	0.47	0.28	0.38
	0.19	0.16	1.17	.040	13.91*	1.07	1.22	0.11	0.53	0.23	1.10	6.40*	0.60	0.51	1.30
	4.26	0.51	2.02	3.49	3.07	2.50	1.98	1.31	1.30	5.81*	1.04	5.10*	4.46*	0.47	1.27
	0.62	0.33	1.82	2.82	9.73*	3.75*	4.77*	4.12	1.35	1.53	1.16	1.40	2.17	0.46	1.31
	0.27	0.57	0.34	0.74	0.97	0.42	1.41	1.05	0.67	0.50	0.96	1.44	0.23	1.43	0.46
	0.16	0.18	1.77	0.67	9.06*	1.14	1.29	0.45	0.47	0.91	0.69	0.48	0.49	0.65	0.33
	3.20	0.78	0.54	1.00	1.62	0.90	0.66	0.20	0.51	0.86	0.55	0.42	0.57	0.52	0.59
Mean (AU)	1.65	1.80	1.16	1.32	0.90	1.03	1.10	1.45	1.21	1.04	1.07	1.17	0.92	1.17	1.28
Standard deviation (SD)	1.32	1.70	0.61	1.01	0.85	0.59	0.57	1.12	0.74	0.63	0.37	0.62	0.53	0.95	0.96
WT ω-3	2.05	0.89	1.08	1.42	0.28	0.99	0.33	1.49	0.15	1.10	1.06	0.44	0.51	1.38	0.39
	2.25	3.54*	2.35	0.97	1.10	0.69	0.34	2.33	1.87	1.48	1.50	1.18	1.15	1.52	1.18
	2.16	4.08*	1.65	1.67	1.13	0.76	0.61	2.42	1.55	2.90*	0.41	0.84	0.78	1.92	0.51
	0.07	0.64	0.29	0.34	0.10	0.26	1.50	0.54	0.27	0.22	0.63	0.36	0.25	0.10	0.62
	1.60	0.81	1.03	0.45	0.41	1.53	0.35	1.21	1.14	0.64	0.81	0.42	0.40	1.77	1.63
	2.28	0.72	1.37	0.38	0.54	1.29	0.32	1.83	2.29	0.61	1.39	0.84	0.66	1.19	2.55
	0.33	0.52	0.39	1.01	0.31	1.08	1.05	1.03	0.47	0.47	0.16	0.86	4.55*	0.60	0.34
	0.19	0.57	1.36	1.57	0.66	1.42	4.36*	2.46	2.02	0.43	1.41	0.72	9.71*	0.48	1.83
	0.09	0.21	0.50	0.95	0.10	1.36	1.00	0.90	0.62	0.50	0.24	2.95*	0.71	0.83	0.36
	0.62	2.31	0.18	0.62	1.55	1.40	2.67	0.72	0.60	0.69	0.12	2.99*	1.09	0.54	0.42
	0.21	0.40	0.43	0.59	0.60	1.34	3.11	0.57	1.02	0.73	0.19	5.18*	7.60*	0.90	0.37
Mean (AU)	1.08	0.59	0.97	0.91	0.62	1.10	1.13	1.41	1.09	0.69	0.72	0.70	0.69	1.02	0.93
Standard deviation (SD)	0.97	0.22	0.68	0.48	0.46	0.39	1.01	0.74	0.74	0.36	0.54	0.28	0.31	0.58	0.76

	Gene expression ($2^{-delta\ delta\ CT}$)														
	Arbitrary units (AU)														
Group	T-bet	IFN-γ	TNF-α	IL-18	IL-2	GATA-3	IL-4	ROR-γ	IL-17	IL-6	TGF-β	FOXP3	IL-10	IL-35	GLUT-1
GK CT	3.45	1.58	8.17	3.18	0.84	0.26	1.03	5.31	6.33	8.86	0.79	0.30	0.46	0.51	2.13
	3.36	12.29	0.32	1.02	1.89	0.16	0.73	3.88	3.27	2.81	1.61	1.49	2.21	0.34	0.75
	3.12	11.44	1.51	2.88	1.65	0.42	0.42	1.59	5.54	5.08	2.71	0.41	0.86	0.11	3.53
	6.70	19.69*	0.49	10.92	1.17	0.18	0.62	1.90	2.31	3.16	1.55	0.75	0.86	0.49	3.17
	6.08	2.10	3.09	1.07	1.08	0.81	0.12	1.95	1.57	0.83	3.97	0.46	1.20	0.18	2.60
	2.35	4.66	1.22	2.93	1.67	1.30*	0.48	7.16	2.49	0.81	1.47	0.81	1.31	0.68	1.98
	2.05	2.18	1.24	2.65	1.41	0.46	1.12	2.97	2.13	3.18	2.46	0.51	1.10	0.55	4.83
	3.14	6.73	1.64	0.97	0.69	0.17	0.16	2.76	2.19	3.04	2.13	0.13	0.07	0.46	3.85
	11.80	4.56	16.62	12.43	26.84*	1.55*	0.69	13.89*	6.55	5.82	10.00*	0.80	5.50*	0.54	2.95
	0.86	1.54	0.67	0.38	7.07	0.08	0.04	2.83	0.67	0.95	1.60	0.36	0.19	0.24	1.39
	2.20	3.88	1.09	3.13	24.95*	0.18	0.22	4.61	1.02	2.37	5.96	0.35	0.11	0.07	4.56
	3.22	2.27	4.44	23.06*	4.69	0.55	0.29	15.10*	1.73	0.72	4.03	0.34	0.23	0.55	2.48
	10.95	7.42	6.16	5.63	4.95	0.43	0.98	3.75	2.70	3.19	5.46	2.80*	2.22	0.44	3.27
	9.38	10.42	18.82*	7.56	41.12*	0.53	0.94	13.19	3.43	10.67	11.77*	1.66	1.84	0.77	3.78
Mean (AU)	4.90	5.47	2.50	5.14	1.30	0.35	0.56	3.52	3.00	3.68	2.81	0.64	0.97	0.42	2.95
Standard deviation (SD)	3.52	3.86	2.50	4.43	0.43	0.22	0.37	1.67	1.87	3.02	1.67	0.46	0.77	0.21	1.16
GK ω-3	0.06	1.47	0.64	0.48	0.18	0.79	0.19	0.29	0.24	1.60	1.52	1.65	1.14	1.42	0.49
	0.21	3.72	0.86	1.10	0.36	0.99	0.31	2.30	0.40	1.81	2.44	1.31	1.84	2.52	0.53
	0.52	1.11	1.25	2.39	0.58	2.54	0.31	2.33	0.88	4.55	1.27	1.05	2.99	3.19	0.97
	1.12	6.52*	1.23	0.68	0.60	0.69	0.17	2.12	0.61	0.77	0.71	1.79	1.39	1.03	1.51
	2.94*	0.40	1.00	0.54	0.78	0.91	0.33	2.67	0.69	2.57	1.36	2.09	3.02	1.86	2.15
	1.64	3.75	0.26	4.03	0.29	0.99	0.35	2.52	0.76	1.38	1.63	0.77	1.33	0.87	2.49
	3.61*	7.29*	4.65*	1.62	0.66	0.63	0.30	3.46	0.65	0.99	0.12	0.72	1.72	1.99	0.63
	0.15	1.99	0.46	1.20	0.92	1.26	2.57*	1.65	3.11	0.54	2.56	1.43	12.32*	1.09	1.68
	0.70	0.25	0.09	1.10	0.44	0.79	0.74	3.67	1.55	0.45	0.66	1.52	0.39	2.91	0.76
	0.61	1.84	0.49	0.86	0.15	0.68	1.04	0.63	1.81	0.47	2.19	0.58	1.94	4.70	2.07
	0.85	0.57	0.42	3.42	1.70	1.77	1.19	1.25	4.85*	0.57	1.69	3.85	8.60*	5.48	1.92
	0.92	0.74	0.52	6.85*	0.84	2.61	3.52*	5.08	6.01*	7.36*	1.88	1.82	2.76	1.74	1.74
	0.33	0.21	0.36	2.89	0.83	2.23	1.04	2.84	3.31	2.65	0.29	3.03	2.54	3.71	1.13
	0.34	0.09	0.14	0.60	0.14	0.48	0.51	0.13	0.97	0.09	0.74	0.85	3.31	1.86	0.29
	0.10	1.77	0.53	0.68	0.09	0.32	0.36	0.81	1.08	0.04	0.46	0.63	4.15	1.54	0.49
Mean (AU)	0.58	1.38	0.59	1.02	0.57	1.18	0.53	2.12	1.24	1.32	1.30	1.54	2.19	2.39	1.26
Standard deviation (SD)	0.46	1.23	0.37	0.57	0.42	0.75	0.35	1.37	0.98	1.25	0.78	0.92	1.04	1.37	0.72
